# Topological variety and self-sorting in homo- and heteroleptic Pd_*n*_L_2*n*_ metallo-supramolecular assemblies

**DOI:** 10.1039/d5sc03203b

**Published:** 2025-06-12

**Authors:** Laura Neukirch, Guido H. Clever

**Affiliations:** a Department of Chemistry and Chemical Biology, TU Dortmund University Otto-Hahn-Str. 6 44227 Dortmund Germany guido.clever@tu-dortmund.de

## Abstract

A plethora of nanoscale Pd_*n*_L_2*n*_-type architectures has been synthesized through the coordination-driven self-assembly of Pd(ii) ions and organic bis-monodentate bridging ligands. While initially, the focus was on homoleptic structures, comprising one type of ligand per assembly, the field has recently shifted towards reducing symmetry in heteroleptic multicomponent assemblies, containing two or more distinct ligands in defined positions. In parallel, the incorporation of functional moieties such as binding and catalytic sites, photoswitches and redox units has seen a steep development. While empirical data have been gathered on the relationship between the ligand structure and assembly outcome for a limited number of cases, confidently forecasting the result of reacting a given ligand with Pd(ii) cations often still remains challenging and has been mastered only for the simplest systems. Additionally, new Pd_*n*_L_2*n*_ topologies – along with subtle factors driving their formation (such as counter anion or guest templation and solvation effects) – are discovered continuously. For designing metallosupramolecular assemblies for application, it is of pivotal importance to increase predictabilty and gain control over assembly topology, as the structure and properties are often closely connected. To raise awareness for the problem's complexity, we commence this review by exploring the surprising breadth of topological diversity among homoleptic Pd_*n*_L_2*n*_ (*n* = 2–8) architectures that has so far been found experimentally. We next discuss strategies for increasing the structural complexity even further through the non-statistical self-assembly of heteroleptic cages, the orientational self-sorting of asymmetric ligands, and chiral self-sorting effects. Special emphasis will be placed on factors governing the particular self-assembly outcome as well as on rationalization approaches based on computations or geometrical considerations.

## Abstract 2

This review covers the vast topological diversity of self-assembled Pd_*n*_L_2*n*_ architectures as well as strategies for the non-statistical synthesis of multicomponent cages by integrative, orientational, and chiral self-sorting.

## Graphical abstract

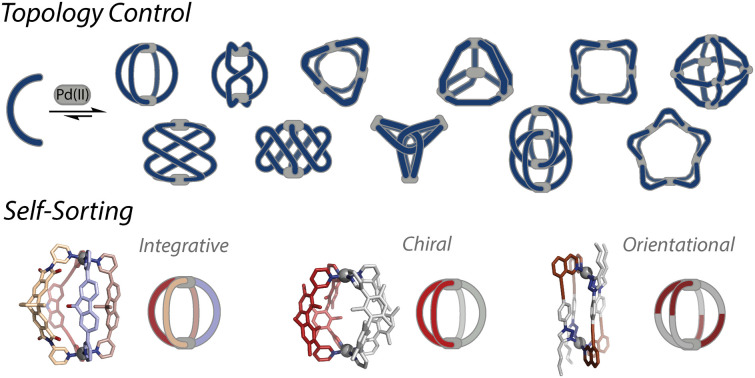

## 1. Introduction

Since the beginning of the field of supramolecular chemistry, researchers have aspired to understand and mimic the high selectivity and specificity of structure formation, binding processes and catalysed reactions found in natural systems.^1^ A closer look into the nanosized cavities of enzymes reveals their often highly unsymmetric, multi-functionalized and dynamic nature.^2^ While imitating certain aspects of the structural and functional complexity of natural systems by means of classical covalent synthesis is possible in principle,^3,4^ such approaches usually involve resource-consuming multi-step organic conversions and tedious purification tasks. The concept of dynamic self-assembly represents an attractive alternative to this: the combination of suitable molecular building blocks with a pre-programmed connectivity allows for the formation of large and intricate supramolecular architectures, usually under thermodynamic or sometimes kinetic control.^5^ Supramolecular assembly approaches can be classified according to the interactions that drive recognition and binding between the different building blocks. Popular approaches are based on dynamic covalent reactions,^6–9^ hydrogen bonding,^10–13^ and metal coordination.^14–22^ In the latter case, metal ions are combined with organic building blocks equipped with a set of suitable donor groups, serving as ligands to bridge two or more metal nodes. Often, transition metal cations with defined coordination geometries are used to enhance predictability of the self-assembly outcome. This approach allows for the construction of extended structures such as coordination polymers or metal organic frameworks (MOFs) as well as discrete, soluble entities such as defined rings, helicates and cages. This review only covers the latter class of non-polymeric compounds. Pioneering examples of such supramolecular coordination architectures include Saalfrank's Mg(ii)-based adamantoid assembly from 1988,^23^ Lehn's helicates,^24^ Raymond's Ga(iii) catecholate tetrahedra,^25^ and Stang's Pt(ii)-based rings and cages.^26^ As a very versatile metal node, Pd(ii) was found to assemble with nitrogen-containing heterocyclic donors such as pyridines and isoquinolines to form defined supramolecular architectures. Aside from the fixed square-planar coordination geometry of d^8^-configured Pd(ii) cations, their diamagnetism (beneficial for NMR analytics), suitable ligand exchange kinetics and the thermodynamic stability of their *N*-ligand complexes render them highly attractive for realizing a large variety of metal-mediated assemblies.^16^

In the early 1990s Makoto Fujita pioneered the construction of two- and three-dimensional assemblies based on *cis*-protected Pd(ii) cations and organic bridging panel-shaped ligands.^20,27^ In 1998, McMorran and Steel reported the first self-assembly of a helical Pd_2_L_4_ cage by employing a Pd(ii) salt with a non-coordinating hexafluorophosphate counter anion and bridging bis-pyridyl ligands with a flexible backbone structure.^28^ Since these initial studies, interest in constructing and applying such Pd(ii) assemblies blossomed.^21,29–31^ Incipiently, the focus of the field was mainly on reporting the successful design or serendipitous finding of new coordination assemblies, largely driven by their aesthetic appeal and the quest for unprecedented topologies. In the last decade, the field transitioned towards implementing functionality into coordination cages, thus bringing them closer to application. In this direction, strategies for modular multicomponent self-assembly started to be developed, allowing the arrangement of several different organic building blocks in close proximity without the need for extensive organic synthesis. Examples for functions that have been incorporated into Pd_*n*_L_2*n*_ assemblies include catalytic activity,^32–37^ light harvesting chromophores,^38^ fluorophores,^39–42^ (photo)-redox activity,^43–45^ photoswitches,^46–50^ and solubility-controlling groups for higher-order self-assembly.^51–53^ Furthermore, many assemblies possess a cavity in which guest molecules can be encapsulated, allowing them to serve as molecular reaction vessels^32,54–56^ and high-affinity receptors,^57–59^ among other functions.^60^ However, most architectures reported so far have high symmetry, sharply contrasting with the low symmetry environments found in natural host systems.^61–63^ Therefore, efforts have been devoted to lowering the symmetry of such artificial self-assemblies by the controlled use of (I) matching organic ligands to form heteroleptic cages,^21,64^ (II) asymmetric ligands,^30^ and (III) different metal-ions to form heteronuclear assemblies.^65^ Self-sorting has also been investigated in the context of organic cages.^66^

**Scheme 1:**
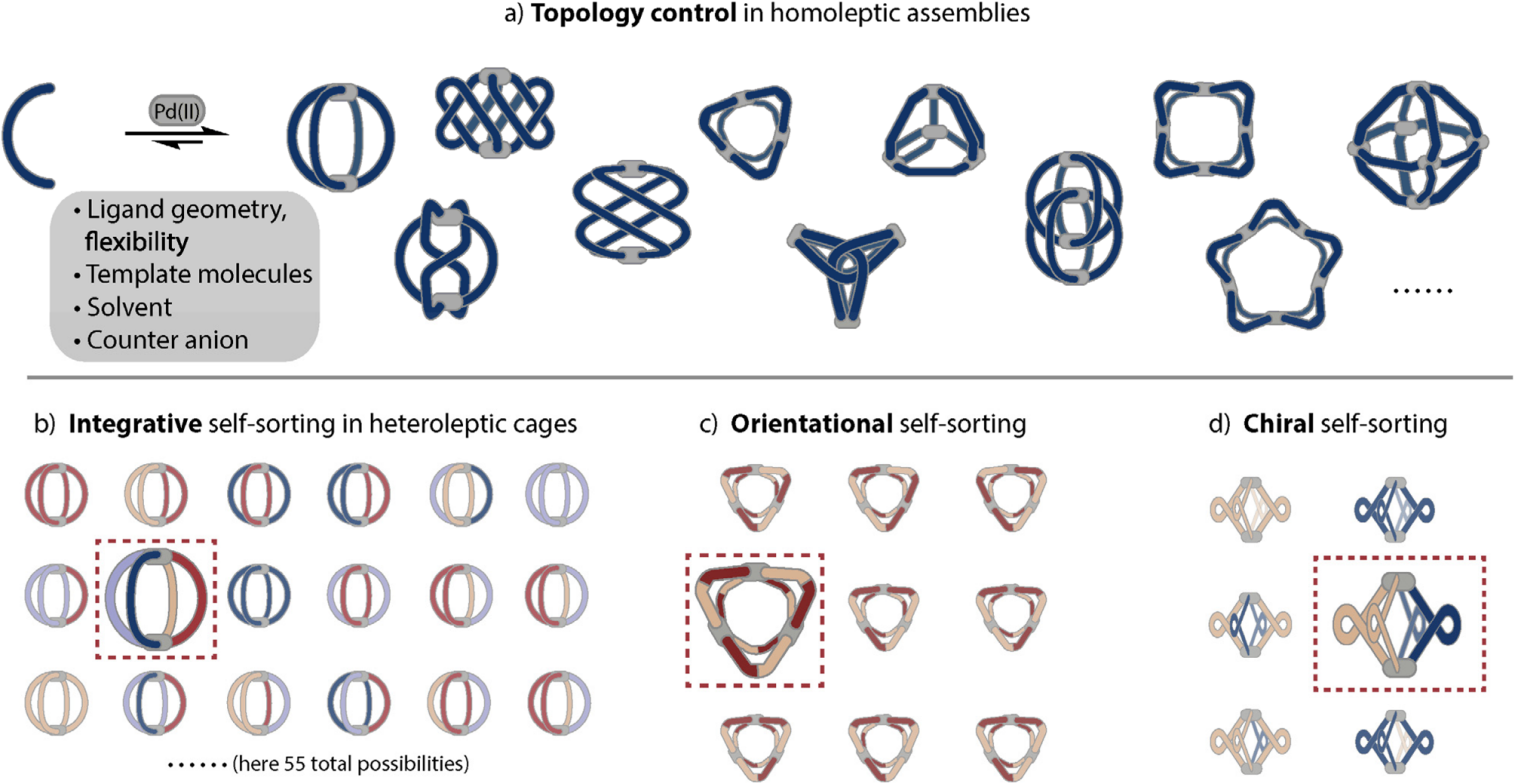
Emergence of structural diversity in Pd_*n*_L_2*n*_ coordination assemblies. (a) Examples for homoleptic architectures with different topologies and nuclearities (the inset shows some factors that may influence the outcome). (b) Integrative self-sorting to one defined heteroleptic cage out of numerous possible dinuclear structures based on four different ligands. (c) Orientational self-sorting to one isomer of a three-membered ring from an asymmetric ligand. (d) Self-sorting of a racemic mixture of chiral ligands to a single cage isomer.

Despite vast strides being made in this field, many of the rules governing the formation of specific and sometimes surprising topologies – often closely connected to the functionality of the assemblies – still remain elusive. Thus, we begin this review by exploring the various topologies found for Pd_*n*_L_2*n*_ homoleptic assemblies, *i.e.* architectures constructed from only one kind of organic ligand (Scheme 1a). While self-assembly is an equilibrium reaction, for simplicity, we use simple arrows throughout the remaining review. Next, we set out to summarise strategies developed for achieving integrative self-sorting to defined heteroleptic assemblies (Scheme 1b). We will then discuss approaches for orientational self-sorting to form homoleptic cages based on asymmetric ligands (Scheme 1c). Lastly, we describe the formation of cages *via* chiral self-sorting phenomena (Scheme 1d).

While there are many reports on assemblies with *cis*- or *trans*-protected Pd(ii) cations and assemblies with ligands of higher denticity^15,21^ as well as on heteronuclear cages,^65^ we limit our discussion here to architectures based on bis-monodentate organic ligands and unprotected Pd(ii) cations. We start this review by highlighting the breadth of topological diversity among homoleptic Pd_*n*_L_2*n*_ (*n* = 2–8) architectures. We then discuss strategies for the non-statistical self-assembly of heteroleptic cages, followed by orientational self-sorting of asymmetric ligands and chiral self-sorting. Special emphasis will be placed on factors governing the particular self-assembly outcome and attempts to rationalize experimental results based on computations or geometrical considerations.

## 2. Homoleptic dinuclear assemblies (*n* = 2)

The most basic and best explored family member among Pd_*n*_L_2*n*_ cages consists of two Pd(ii) ions bridged by four organic ligands. After the initial work on a quadruple stranded helicate Pd_2_L_4_ by McMorran and Steel,^28^ a multitude of cages and helicates has been reported, with many studies focusing on the creation of larger cavities^67,68^ for the encapsulation of big^58,69,70^ or multiple^58,71,72^ guests and/or towards the incorporation of functionalities in the ligand backbone.^38–40,42–47,50,51^ As the field of the so-called lantern-shaped Pd_2_L_4_ cages has been reviewed recently,^29^ we will only briefly discuss some key parameters governing their formation and highlight prominent examples. Some helicates will be tackled with special emphasis on the degree of helicity. Finally, we describe in more depth some recent examples for some rather sophisticated dinuclear topologies, including those that do not obey the principle of maximum site occupancy, *i.e.* assemblies of stoichiometry Pd_2_L_2_ and Pd_2_L_3_.

### 2.1. Lantern-shaped cages and helicates

**Fig. 1:**
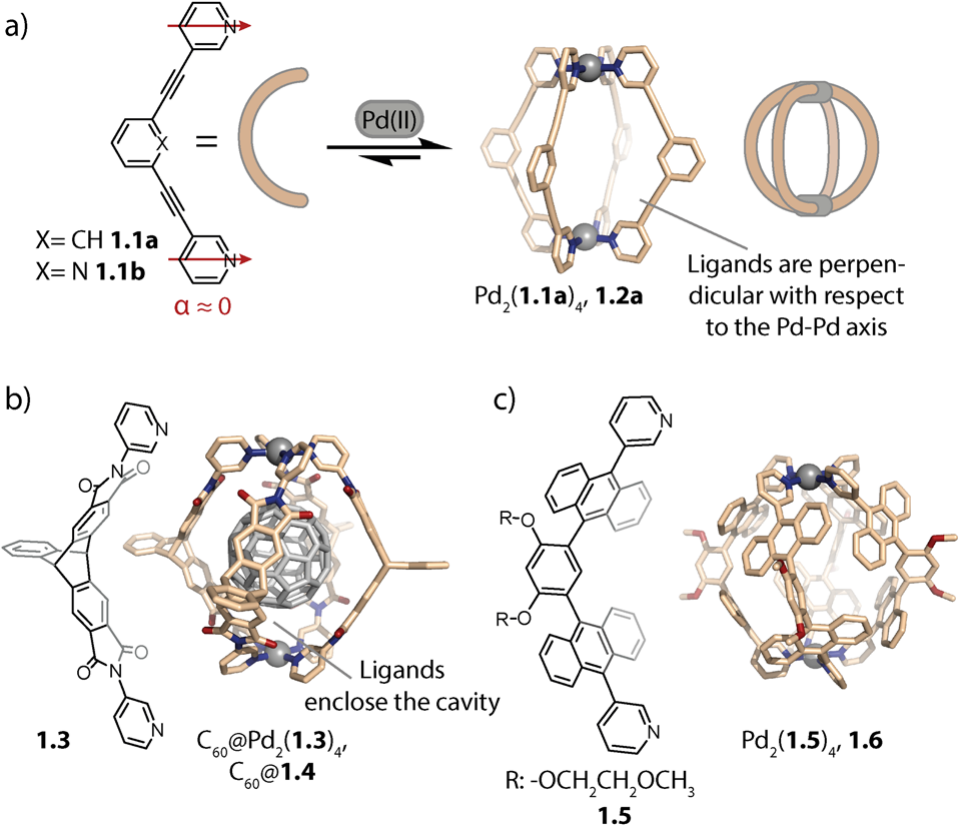
(a) Self-assembly of lantern-shaped cage 1.2a. (b) Capsule 1.4 based on a triptycene-derived ligand, encapsulating a C_60_ fullerene. (c) Capsule 1.6 based on ligands with anthracene panels.

The geometry of rigid bispyridyl-type bridging ligands can be described through the binding angle *α*, defined as the angle between the two nitrogen donor vectors. For ligands with collinear vectors, so-called lantern-shaped cages are preferably formed (Fig. 1a). A prominent example for a lantern-shaped cage is Pd_2_(1.1a)_4_, (note the naming scheme used herein: ligand 1.1a reacts to form cage 1.2a), initially reported by Hooley and coworkers in 2010.^73^ Here, the π-surfaces of the ligands are oriented in a perpendicular fashion with respect to the longer axis of the cavity (the latter going through both Pd centres), allowing for inward-pointing functionalities to be placed in the backbones. Lusby and coworkers exploited the inward pointing α-protons of the coordinating pyridines for forming multiple hydrogen bonds to the oxygens of quinones, resulting in encapsulation with high association constants.^74^ Furthermore, the same group used 1.2b for efficiently catalysing cycloadditions to the thus activated substrates inside the cage cavity.^33–35^

On the other hand, ligands with aromatic surfaces that partially enclose the cavity have been employed to assemble Pd_2_L_4_ capsules. For example, we designed curved ligand 1.3 based on triptycene^58,75^ (and ligands with a similarly shaped backbone^76^) aiming at a size match between the cavity of the corresponding capsule Pd_2_(1.3)_4_, 1.4 and fullerene C_60_ (Fig. 1b). This allowed selective binding of C_60_ and thereby its solubilization in polar organic solvents.

Yoshizawa and co-workers reported capsule Pd_2_(1.5)_4_, 1.6 based on ligand 1.5, whose methoxy ethoxy substituents endow the assembly with good solubility, even in water (Fig. 1c). The cavity of 1.6 is surrounded by an array of anthracene panels, creating a hydrophobic confinement. Owing to these two key properties, capsule 1.6 is capable of binding a range of neutral guests in aqueous solution.^59^ Recently, the same group used a similar capsule decorated with sugar substituents to induce chirality on an encapsulated fullerene.^77^ Our group, in collaboration with Gunnlaugsson and coworkers, has also achieved chirality transfer onto fullerenes (C_60_ and C_70_) with a readily accessible, relatively lightweight Pd_2_L_4_ cage with ligands constructed from Tröger's base.^78^

**Fig. 2:**
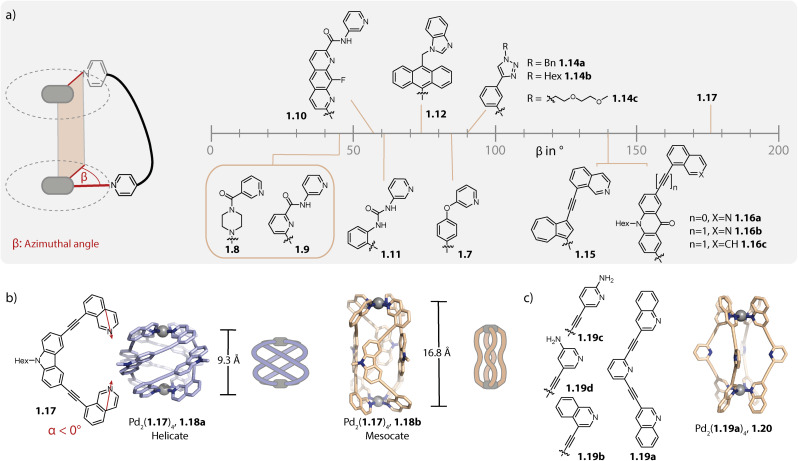
(a) Overview of azimuthal angles *β* of some Pd_2_L_4_ helicates formed with different bis-monodentate organic ligands (the *β* values were taken from the corresponding publication when available or were determined based on the X-ray crystal structure as described in ref. 96). (b) Structure of helicate 1.18a in comparison to isomeric mesocate 1.18b. (c) Structure of ligands 1.19 and mesocate 1.20.

Certain factors such as the ligand geometry,^38,79,80^ steric congestion at the backbone^81^ or donor site,^82^ and attractive inter-ligand interactions^83,84^ can favour a helical twisting of the ligands around the Pd–Pd axis. For characterizing the degree of helicity, the azimuthal angle *β* can be used. It is defined as the dihedral angle between the two N–Pd coordination bonds of the same ligand (Fig. 2a).

In their initial report, McMorran and Steel found one PF_6_^−^ anion encapsulated in the cavity of helicate Pd_2_(1.7)_4_ which is based on flexible ligand 1.7 (Fig. 2a).^28^ This recently enticed the authors to study helicate formation in the presence of a variety of counter anions. Tetrahedral anions were found to be preferred over octahedral PF_6_^−^ and amongst the anions tested, ClO_4_^−^ showed the strongest binding, probably due to a perfect size match. On decreasing the size of the anions, the assembly shrinks along its Pd–Pd axis, concomitant with an increase in helicity, enabling an almost constant degree of cavity filling. The largest azimuthal angle of 85° was observed with iodine.^85^

Chand and coworkers reported in 2010 on helicate Pd_2_(1.8)_4_ consisting of piperazine-based ligand 1.8. The ligand adopts a flattened *anti* conformation upon self-assembly and the helicate is characterized by an azimuthal angle of around 45°.^86^ In 2012, the same group reported a helicate with a similar azimuthal angle by employing another flexible ligand 1.9 based on a central pyridine (not participating in metal coordination) that was equipped with amide linkers.^87^ The ligand can adopt different conformations depending on the orientation of the amide linkages relative to the backbone. It was found that the carbonyl bonds rotate outside of the helicate, presumably to avoid electronic repulsion with the central pyridine nitrogen and for enabling H-bonding between the NH groups and the encapsulated anion. In contrast, the same ligand with a benzene core formed, albeit under different conditions, a lantern-shaped cage, as reported by Puddephatt and coworkers in 2004. In this case, one of the two carbonyl groups of each ligand indeed points inside the cavity.^88^

In 2018, Gan and coworkers showed that ligand 1.10 assembles to form a mixture of *D*_4_-symmetric helicate and *C*_4h_-symmetric mesocate when combined with Pd(NO_3_)_2_. Selective formation of helicate or mesocate was favoured with a B_12_F_12_^2−^ guest or with tetrafluoroborate, respectively. Furthermore, addition of the chiral anion Δ-TRISPHAT led to an enrichment of one of the helical enantiomers, as the authors showed by CD spectroscopy.^89^

Additionally, in 2021, Chand and coworkers reported the anion-controlled self-assembly of helicate *versus* lantern-shaped cages: ligand 1.11 with urea linkers yields non-helical Pd_2_(1.11)_4_ in the presence of NO_3_^−^ while a helicate with an angle of approximately 60° is formed with ClO_4_^−^ anions.^90^

Sun and coworkers reported in 2015 on helicate Pd_2_(1.12)_4_ comprising anthracene-based ligands 1.12 equipped with benzimidazole donor moieties. Ligand twisting, resulting in a *D*_4_-symmetrical helicate with an azimuthal angle of around 75°, can be traced back to the steric repulsion between the anthracene backbones. The latter creates a hydrophobic cavity which encapsulates a nitrate anion that is stabilized by multiple H-bonds to the acidic imidazole protons. The authors showed that the binding affinity towards nitrate is significantly higher as compared to those of other anions tested.^81^

Crowley and coworkers reported helicate Pd_2_(1.14a)_4_ based on a small triazine-terminated ligand modified with benzyl groups. The helicate is further stabilized by π–π interactions and the azimuthal angle reaches around 90°.^91^ In a follow-up study, the group modified the triazine appended moiety as well as the spacer. For the latter, the helicates were shown to form reliably when the 1,3-phenylene moiety was substituted with a flexible propyl chain but not when substituted with a 1,4-phenylene group. Furthermore, the helical structure displayed tolerance across all tested exohedral triazine substituents, ranging from alkyl chains to electron-rich and -poor aromatic groups.^92^ In the context of biological activity, the Crowley and Giles group studied the stability of Pd_2_(1.14a–c)_4_ (Fig. 2a) and 1.2b (see Fig. 1a) against biological nucleophiles. Here, the helicates showed higher stability compared to lantern-shaped cage 1.2b, presumably due to the increased donor strength of the triazine and the sterically shielded Pd(ii) centres. Furthermore, Pd_2_(1.14b)_4_ proved more stable compared to Pd_2_(1.14a)_4_ and Pd_2_(1.14c)_4_, originating most likely from the more restricted access to the coordination sites, owing to agglomeration of the alkyl chains.^93^

We have reported the self-assembly of helicates from various ligands possessing 8-isoquinoline donor moieties. The choice of this donor moiety leads to strongly convergent bonding vectors and hence, the ligands have to severely twist for bridging the two Pd(ii) ions. The helical arrangement is further stabilized by π–π interactions between the ligands. Self-assembly of ligands 1.15 (ref. 79) and 1.16a, 1.16b (ref. 80 and 94) which are based on azulene and acridone backbones, respectively, led to helicates with azimuthal angles of around 140°. We also synthesized helicates based on well-known coal-tar dyes; in this case, piperazine linkers were used, allowing for conserving the absorption properties of the dyes.^38^ The corresponding ligands based on acridone, azulene, or coal-tar dyes equipped with 3-pyridyl donors instead of 8-isoquinolines assemble to form interlocked double cages^95^ or to lantern-shaped cages,^38,79^ respectively. This can be traced back to the nearly collinear binding vectors of the latter, showcasing how the topology of the dinuclear species in terms of its helical twist can be controlled with the ligand geometry.

In order to attain even greater azimuthal angles, we recently investigated the self-assembly of carbazole-based ligand 1.17 with an even more negative binding angle. Formation of helicate Pd_2_(1.17)_4_, 1.18a was observed with various different Pd(ii) salts and the azimuthal angles ranged between 171 and 176° (Fig. 2b). In each case, one anion was found to be encapsulated within the cavity. Interestingly, with an increase in the size of anion, the Pd–Pd distance decreased, concomitant with a widening of the cage *i.e.* a change in the shape from prolate to oblate. While this might sound contradictory to the earlier report of McMorran and Steel,^85^ the structural adaptation serves the same purpose, that is to retain a favourable cavity filling. Helicate 1.18a exists in a counter anion- and solvent dependent equilibrium with the corresponding mesocate 1.18b. In the latter, the ligands adopt *C*_s_ symmetry, resulting in a significantly larger Pd–Pd distance (16.8 Å as compared to 9.3 Å) and an overall achiral structure.^96^

Crowley and coworkers modified ligand 1.1b by either exchanging the pyridine with quinoline or isoquinoline donors (1.19a and 1.19b)^83^ or by equipping the pyridines with amino groups at *ortho*- or *meta*-position to the nitrogens (1.19c and 1.19d).^97^ Ligands 1.19b and 1.19d form, similarly to parent ligand 1.1b, a lantern-shaped cage. However, self-assembly of 1.19a and 1.19c which possess modifications in closer proximity to the coordinating nitrogen resulted in the formation of a mesocate Pd_2_(1.19a)_4_, 1.20 and a helicate Pd_2_(1.19c)_4_ (Fig. 2c). For Pd_2_(1.19c)_4_, this was explained by hydrogen bonds between the amino groups in the twisted coordination environment.

### 2.2. Unusual dinuclear assemblies

**Fig. 3:**
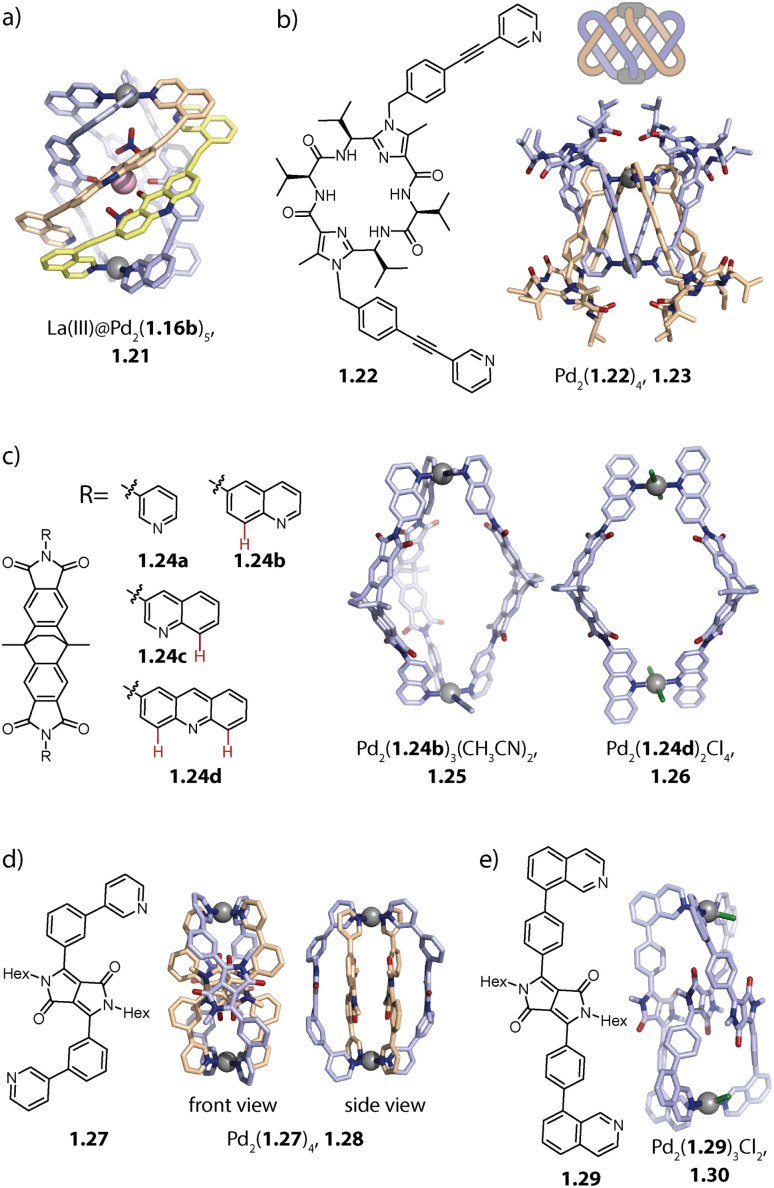
(a) Penta-stranded helicate 1.21 templated by La(iii). (b) Doubly interlocked lemniscates 1.23. (c) Bowl 1.25 and ring 1.26 (protons marked red would cause steric clash in a potential Pd_2_L_4_ arrangement). (d) Double-bridged ravel 1.28. (e) Single-bridged ravel 1.30. Positions on Pd(ii) centres not saturated by N-heterocyclic ligands are either occupied by solvent or halide ligands.

Severin and coworkers observed that acridone-based helicate Pd_2_(1.16b)_4_, initially reported by us,^80^ interacts with a fifth ligand 1.16b, when Pd(ii) and 1.16b are combined in a 2 : 5 ratio. In order to get insight into the nature of this association, the authors added 1.16c which is a derivative of 1.16b, lacking the nitrogen donors. The authors observed the formation of a 1 : 1 complex between 1.16c and helicate Pd_2_(1.16b)_4_. The association of compound 1.16c with Pd_2_(1.16b)_4_ is presumably driven by strong ligand-to-ligand π–π stacking interactions. For obtaining a discrete penta-stranded helicate Pd_2_L_5_, the authors exploited the multiple inward pointing carbonyl groups of the helicate: oxophilic, high-valency La(iii) binds to the pocket of the helicate and stabilizes the arrangement of five ligands 1.16b*via* coordination bonds. In La(iii)@Pd_2_(1.16b)_5_, 1.21, three ligands coordinate to both Pd(ii) centres while the two remaining ones coordinate *via* one donor group only, giving rise to an assembly of overall low symmetry (Fig. 3a).^84^

Our group obtained a complex, interlocked architecture Pd_2_(1.22)_4_, 1.23 with bis-pyridyl ligand 1.22 which is based on a peptidic macrocycle (Fig. 3b). The assembly is chiral and represents an entirely new catenated motif for Pd(ii) architectures. The high ligand flexibility allows it to chelate to a single metal ion to give mononuclear complex Pd(1.22)_2_, possessing a butterfly structure. Self-assembly in acetonitrile, followed by the addition of chloride or triflimide salts, however, eventually led to the formation of a dinuclear complex. The Pd(ii) centres in the *C*_2_-symmetric structure are chelated in a *cis*-configuration and the two Pd(1.22)_2_ moieties, also termed lemniscates, are held together by two mechanical bonds. A closer look at the X-ray structure reveals that this peculiar architecture is stabilized by (a) a counter anion that binds between the two Pd(ii) centres, (b) inter-ligand π–π stacking interactions and (c) embedding of pyridine donor moieties in the macrocyclic backbone cavity of the adjacent ligand.^98^

Moreover, we showed that the introduction of steric congestion on the donor site can result in the formation of Pd_2_L_*m*_, *m* < 4 species, thereby challenging the principle of maximum site occupancy. While pyridine-equipped ligand 1.24a based on a dibenzo-2.2.2-bicyclooctane backbone assembles to form capsule Pd_2_(1.24a)_4_, the same ligand with 6-quinoline donors 1.24b yields bowl Pd_2_(1.24b)_3_(CH_3_CN)_2_, 1.25 when Pd(ii) and 1.24b are combined in a 2 : 3 ratio (Fig. 3c). This outcome can be explained by steric repulsion: in capsule Pd_2_(1.24b)_4_, the distances between protons ^8^H (red in Fig. 3c) of neighbouring ligands are smaller than the sum of their van der Waals distances, leading to steric repulsion, while they have more space and thus surmount this distance in bowl 1.25.^76^ Interestingly, when ligand 1.24c, equipped with 3-quinoline donors is employed, capsule Pd_2_(1.24c)_4_ is formed as a thermodynamic product. For overcoming steric stress, the donor groups arrange in a propeller geometry, leading to an overall helical twist of the assembly. Acridine as a donor moiety introduces two hydrogen substituents that are oriented in a similar direction to the nitrogen donor vectors. Ligand 1.24d hence has a further increased steric hindrance compared to 1.24b and 1.24c, favouring the formation of ring Pd_2_(1.24d)_2_(CH_3_CN)_4_/(Cl)_4_, 1.26.^82^ This topological variance has consequences for the fullerene binding capabilities of the assemblies. While capsule Pd_2_(1.24a)_4_ is able to bind C_60_ but not C_70_, the open bowl 1.25 allows for binding of both. Helically twisted Pd_2_(1.24c)_4_ and ring 1.26, on the other hand, showed no or weak affinity towards fullerenes. Partial solvent exposure of the surface of C_60_ in bowl 1.25 was exploited for controlling the covalent functionalization of fullerenes with the bowl serving as a non-covalent protecting group.

Formation of Pd_2_L_3_-type bowls along with Pd_2_L_2_-type rings was also observed for bis-picolyl ligands, *i.e.* when the steric congestion at the pyridine is increased through the introduction of methyl groups.^99^ Furthermore, coordination rings are also formed when doubly protected Pd(ii) cations or a moiety such as PdCl_2_ is employed.^100–102^

Recently, we reported a self-penetrated homoleptic assembly based on ligand 1.27 which possesses two key attributes: (a) strongly convergent donor vectors and (b) diketopyrrolopyrrole (DPP) backbones, known for their high propensity to interact *via* π–π stacking. Doubly-bridged ravel Pd_2_(1.27)_4_, 1.28 is obtained as a racemic mixture, belonging to point group *D*_2_. The two inner ligands adopt an S-shaped conformation which allows them to cross the centre of the assembly. The other two ligands sit on the outer positions and are helically twisted (Fig. 3d). Overall, four DPP moieties are stacked whereby neighbouring backbones are rotated by roughly 90°. The assembly is reminiscent of heteroleptic *trans*-Pd_2_A_2_B_2_ cages,^103^ which will be described later. In ligand 1.29 the DPP backbone is equipped with sterically demanding isoquinoline donors which led to the formation of self-penetrated bowl Pd_2_(1.29)_3_, 1.30. In contrast to previously reported bowl structures, the central and one of the outer ligands adopt an S-shaped conformation. This allows three DPP moieties to stack and gives rise to a *C*_2_-symmetric assembly, not leaving any cavity for guest binding (Fig. 3e).^104^

In summary, here we showcased a series of experimentally observed topologies for assemblies possessing only two Pd(ii) ions. The design principles for lantern-shaped cages and helicates are well established. For the former, collinear bis-monodentate ligands are utilized; helical twisting is driven by steric constraints, inter-ligand interactions, and/or strongly convergent donor vectors. We also touched upon examples where the steric demand of the donor groups resulted in the formation of more open bowl or ring structures. It is notoriously more difficult to design intricate self-penetrated or interwoven assemblies whose formation is often governed by close-ligand interactions, templation, solvent effects or a combination thereof.

## 3. Homoleptic multinuclear assemblies (*n* = 3–8)

Dinuclear cages (Chapter 2) and spheres Pd_*n*_L_2*n*_*n* ≥ 12 have garnered great attention and for the latter, guidelines for their rational design have been described by Fujita and coworkers.^105^ Furthermore, most functional assemblies reported so far belong to either of these classes.^64,105^ Less attention has been paid to assemblies with intermediate sizes (*n* = 3–8), which will be described in this chapter. We discuss the assemblies in the order of increasing nuclearity and complexity, starting with three- to five-membered rings. Next, we will discuss pseudo-tetrahedral and hexanuclear species such as cubes, octahedra, and rings. Finally, we address more complex supramolecular coordination assemblies, such as interlocked cages. For clarity, different assemblies based on the same ligand are usually discussed together; hence, the order is not strictly followed.

### 3.1. Coordination rings

**Fig. 4:**
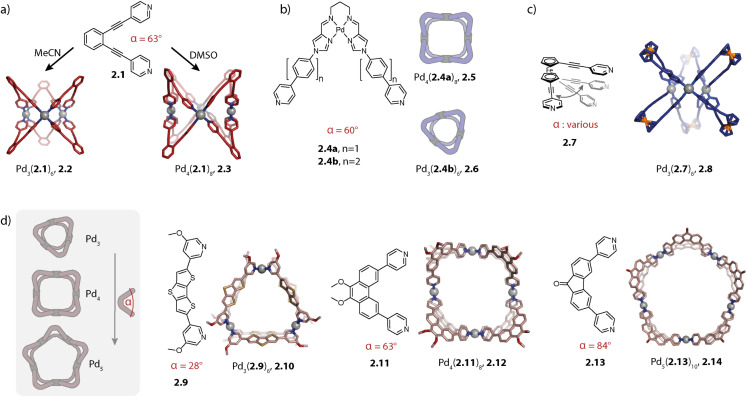
Self-assembly of homoleptic rings Pd_*n*_L_2*n*_ (*n* = 3–5). (a) Solvent-controlled self-assembly of three- and four-membered rings 2.2 and 2.3. (b) Self-assembly of three- and four-membered rings 2.5 and 2.6 controlled by the ligand length. (c) Three-membered ring 2.8 based on a rotationally flexible ferrocene-based ligand. (d) Control over the ring size achieved through ligand angle adjustment (all X-ray structures; for 2.10, a DFT model is shown). For Pd_6_L_12_ rings, see Fig. 5b and 8d (*α* refers to the binding angle of the free ligand and the values for ligands 2.1, 2.4, 2.9, 2.11, and 2.13 were taken from ref. 112).

For the nuclearities *n* = 3, 4, and 5, the assemblies of the highest symmetry are rings. Fujita and coworkers synthesized the first three-membered ring in 2006 which will be described later.^106^ The same group showed that ligand 2.1, possessing a binding angle of 63°, assembles to form the entropically favoured three-membered ring Pd_3_(2.1)_6_, 2.2 in acetonitrile, while a four-membered ring Pd_4_(2.1)_8_, 2.3 is formed in DMSO (Fig. 4a). Interestingly, X-ray crystal structure analysis revealed that the cavity of 2.3 in the solid state is occupied by an aggregate of DMSO and nitrate molecules. Hence, the solvent dependency might be explained by a templation effect. Self-assembly in the presence of other anions yields mixtures of tri- and tetranuclear species, supporting this conclusion.^107^ Previously, a four-membered ring Pd_4_L_8_ had only been reported by Chand, Fujita and coworkers in 2003.^108^ A similar solvent-dependent equilibrium between 3- and 4-membered rings was recently described in mechanistic detail by Li and coworkers.^109^

Previously, the same group also investigated the self-assembly of metalloligands 2.4a and 2.4b with binding angles of 60°, differing solely in their length and hence in their flexibility. While short ligand 2.4a cleanly assembles to form four-membered ring Pd_4_(2.4a)_6_, 2.5, ligand 2.4b forms trinuclear ring Pd_3_(2.4b)_6_, 2.6 (Fig. 4b). Hence, the increased flexibility of ligand 2.4b allows the formation of the entropically favoured smaller assembly.^110^

Crowley and coworkers studied the self-assembly of ferrocene-based ligand 2.7, which can, in contrast to the rather rigid ligands discussed beforehand, adopt a variety of different binding angles owing to its rotational flexibility (Fig. 4c). Interestingly, the three-membered ring Pd_3_(2.7)_6_, 2.8 was obtained as the sole species. Presumably, this is the smallest assembly accessible without significant ring strain; hence, the outcome can be rationalized based on entropic considerations.^111^

We recently studied the self-assembly of a series of ligands under systematic variation of the binding angle *α* under similar conditions (DMSO, [Pd(CH_3_CN)_4_](BF_4_)_2_) for deriving a relationship between the ligand bent angle and ring nuclearity (Fig. 4d). Ligand 2.9 with an angle of 28° assembles to form the three-membered ring Pd_3_(2.9)_6_, 2.10 and phenanthrene-based ligand 2.11 with an angle of 63° forms, as was shown previously,^80^ the four-membered ring Pd_4_(2.11)_8_, 2.12. The binding angle of fluorenone based ligand 2.13 is further increased to 84° which allowed us to cleanly assemble the first non-templated five-membered ring Pd_5_(2.13)_10_, 2.14. Self-assembly of a dibenzothiophene-based ligand with an intermediate binding angle of 78° leads to the formation of a mixture of four- and five-membered rings, showcasing that the ring size can be controlled by the bent angle when self-assembly conditions and ligand flexibility remain constant. It has to be noted that the self-assembly of the discussed ligands displayed solvent dependency and also tetra- and hexanuclear species, most probably octahedra, could be observed by mass spectrometric analysis. By treating the ligands as rigid triangles, a geometrical model was derived which reproduced the observed trends in a qualitative fashion. Major bottlenecks of such simple geometrical considerations, however, are omission of the ligand flexibility and solvent effects, hence serving only as a rule of thumb apporach.^112^

Crowley and coworkers synthesized a heterometallic Pd_3_(PtL_2_)_6_ ring by employing a ligand with a bidentate and two monodentate binding sites. In the first step, Pt-based metalloligand L_2_Pt was synthesized through coordination *via* the bidentate site. This was followed by self-assembly with Pd(ii). When L was reacted with only Pd(ii) cations, a mixture of different assemblies was obtained and Pd_3_(PdL_2_)_6_ was identified as the main component. The heterometallic ring was shown to incorporate planar aromatic guests within its clefts and was used for catalysing the [4 + 2] cycloaddition of anthracene with singlet oxygen.^113^

In 2014 and 2015, the groups of Mukherjee and Chand assembled a three-membered ring Pd_3_L_6_ from simple 1,3-diimidazole-benzene ligands.^114,115^ Chand showed that a similar ligand with benzimidazole donors also assembles to form a three-membered ring and that this assembly allows for gel formation owing to the additional π-surfaces.^115^

Furthermore, we equipped a photoswitchable dithienylethene (DTE) backbone with *para*-pyridine donors. Opening and closing of the DTE moiety upon irradiation with UV or visible light, respectively, is accompanied by a drastic change in the ligand bent angle. This allowed for reversibly switching between a mixture of rings (Pd_3_L_6_ : Pd_4_L_8_ 3 : 1) and a Pd_24_L_48_ sphere. While assembly in the presence of tetrafluoroborate anions afforded the aforementioned mixture of three- and four-membered rings, the trinuclear species was formed as the sole species with a Pd(ii) nitrate salt. Presumably, this can be attributed to a templation effect.^116^

**Fig. 5:**
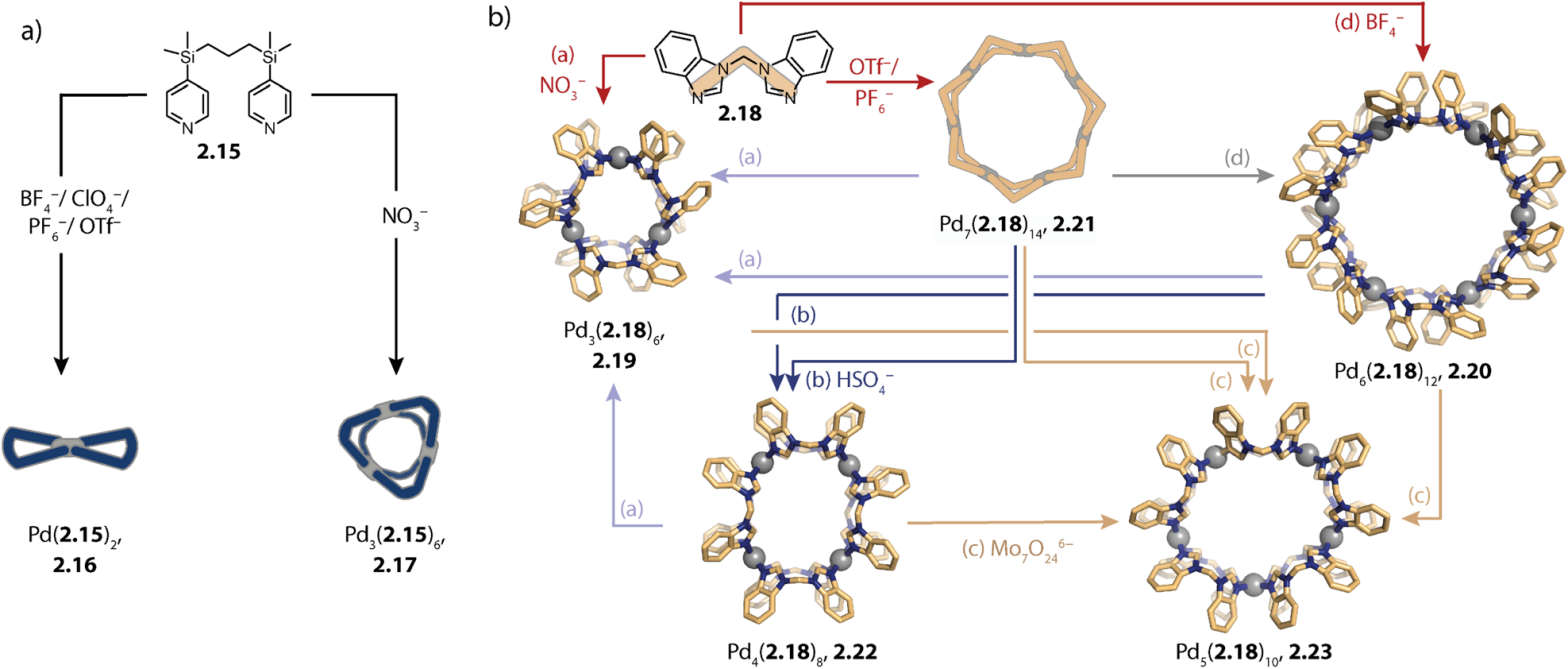
Self-assembly of homoleptic rings Pd_*n*_L_2*n*_ (*n* = 3–7) by templation. (a) Flexible ligand 2.15 self-assembles to form entropically favoured lemniscate 2.16 or *via* templation to the three-membered ring 2.17. (b) Flexible, small ligand 2.18 assembles to form rings 2.19–2.23 of various sizes depending on the counter anion(s) present.

Templation of Pd_3_L_6_ by nitrate anions was also reported by Jung and coworkers. The authors showed that self-assembly of a very flexible bis-pyridyl ligand 2.15 with different Pd(ii) precursors results in entropically favoured lemniscates Pd(2.15)_2_, 2.16. In contrast, when Pd(ii) nitrate was used as the metal source, a clean three-membered ring Pd_3_(2.15)_6_, 2.17 was formed (Fig. 5a).^117^

In a similar fashion, Sun and coworkers showed nitrate templated formation of the three-membered ring Pd_3_(2.18)_6_, 2.19 (Fig. 5b). Surprisingly, rings of various nuclearities, ranging from *n* = 3–7 were assembled with ligand 2.18, strictly depending on the anion(s) present in solution. Akin to the three-membered ring, the six- and seven-membered rings Pd_6_(2.18)_12_, 2.20 and Pd_7_(2.18)_14_, 2.21 were obtained through direct assembly with the BF_4_^−^, or PF_6_^−^/OTf^−^ salts, respectively (red arrows in Fig. 5b). In contrast, four-membered ring Pd_4_(2.18)_8_, 2.22 could only be accessed *via* ring-to-ring transformation by addition of HSO_4_^−^ to preformed 2.20 or 2.21 (dark blue arrows) and five-membered ring Pd_5_(2.18)_10_, 2.23 by addition of Mo_7_O_24_^6−^ to any of the preformed rings (beige arrows). From the different transformation processes, the authors concluded that the templation effect for this system is decreased in the order Mo_7_O_24_^6−^ > NO_3_^−^ > SO_4_^2−^ > BF_4_^−^ > PF_6_^−^ = OTf^−^. The high adaptivity was attributed to (a) the methylene group which enables rotation as well as bending and hence adoption of numerous binding angles and (b) the acidic CH protons which might contribute to specific anion binding.^118^

### 3.2. Tetrahedra

**Fig. 6:**
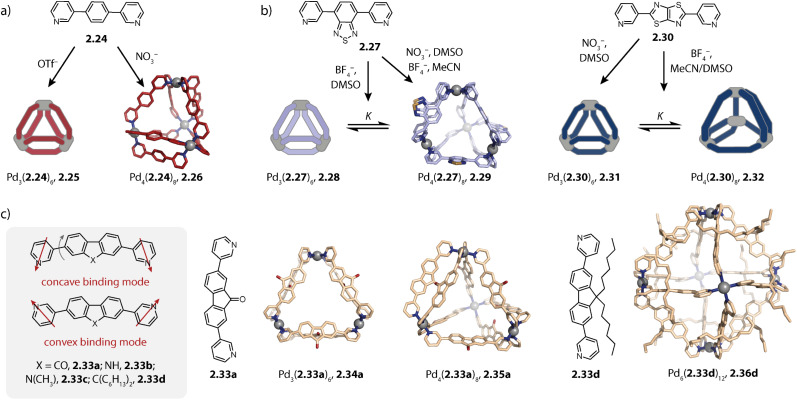
Self-assembly of Pd_3_L_6_ rings and Pd_4_L_8_ pseudo-tetrahedra with bis-*meta*-pyridyl ligands. (a) Counter-anion dependent self-assembly of 2.25 and 2.26. (b) Self-assembly of three-membered rings 2.28 or 2.31 and tetrahedra 2.29 or 2.32, dependent on the counter anion and solvent. (c) Fluorene-derived ligands 2.33 can adopt different binding modes, allowing for assembly to Pd_3_L_6_ rings, Pd_4_L_8_ tetrahedra, and Pd_6_L_12_ octahedra.

In contrast to *para*-pyridine-based ligands which often form three-membered and/or four-membered rings,^107,110,112^ small ligands equipped with *meta*-pyridine donors tend to assemble to form pseudo-tetrahedra as tetranuclear species.^106,119,120^ The first example of a tetrahedron Pd_4_L_8_ was reported by Fujita and coworkers in 2006. The authors showed that short ligand 2.24 forms a triangular species Pd_3_(2.24)_6_, 2.25 with triflate as the counter anion while a tetrahedron Pd_4_(2.24)_8_, 2.26 was obtained when a nitrate salt was employed (Fig. 6a). Note that Pd_4_L_8_ assemblies can only give rise to pseudo-tetrahedral structures in which two oppositely arranged edges are doubly bridged while the remaining four edges are bridged by a single ligand each. Hence, each vertex consists of a tetra-coordinated Pd(ii) cation (whereas in a real tetrahedron, each vertex connects only three edges). Elongation of the ligand with one phenyl linker results in a dynamic equilibrium between both species: with nitrate, the triangular ring and the tetrahedron coexist in a ratio of 1 : 1 while the ring is favoured when a Pd(ii) triflate salt is employed. With tosylate, the tetrahedral species is yielded as the sole assembly.^106^

Mukherjee and coworkers studied the self-assembly of small bis-*meta*-pyridyl ligands of a similar geometry. Self-assembly of ligand 2.27 with a Pd(ii) nitrate salt yielded, similar to 2.24, tetrahedral cage Pd_4_(2.27)_8_, 2.29 exclusively. In contrast, ligand 2.30 assembles to form triangular Pd_3_(2.30)_6_, 2.31 under the same conditions (Fig. 6b). The self-assembly of ligand 2.27 was furthermore studied with Pd(ii) tetrafluoroborate salt in DMSO and ACN. While the tetrahedral species 2.29 was favoured in ACN with this counter anion as well, a mixture of ring 2.28 and tetrahedron 2.29 was obtained in DMSO. Self-assembly of ligand 2.30 yielded a dynamic mixture in both solvents with the triangular species 2.31 as the main component in ACN and tetrahedral 2.32 in DMSO. From the investigation of the equilibrium of both DMSO systems at various temperatures, the authors were able to conclude that the tetrahedra 2.29 and 2.32 are entropically favoured over the corresponding rings but are enthalpically disfavoured; hence, the equilibrium shifts towards the larger species at higher temperatures.^120^

Moreover, our lab investigated the self-assembly of fluorene-derived ligands 2.33.^119,121^ Considering the flat ligands, two binding situations, namely the concave and the convex mode, can be differentiated (Fig. 6c). A binding mode in which the two donor vectors are oriented in opposite directions would most probably not lead to a discrete assembly. Fluorenone-^119^ and *N*-methyl carbazole^121^ based ligands 2.33a and 2.33c assemble to form a mixture of the three-membered ring Pd_3_(2.33a/c)_6_, 2.34a/c and tetrahedral Pd_4_(2.33a/c)_8_, 2.35a/c in acetonitrile. In contrast, ligand 2.33b based on *N*-unsubstituted carbazole cleanly assembles to form the tetrahedral species 2.35b. Even though the ligands in the assembly are not flat, the two distinct binding modes are observed in the X-ray crystal structure of 2.35a (Fig. 6c, middle): the ligands sitting on the singly bridged edges adopt a rather convex binding mode, and hence the backbone functionality points outside, while the ligands occupying the doubly bridged edges adopt a concave binding mode. When fluorene-based ligand 2.33d with two hexyl chains is employed, octahedral Pd_6_(2.33d)_12_, 2.36d is obtained. All ligands adopt a convex binding mode, allowing for assembly to this higher nuclearity species. Occupation of double-bridged edges is not possible with 2.33d owing to steric hindrance; hence, formation of homoleptic rings and tetrahedra is impeded.^119^ It is worth noting that the backbone steric bulk does serve as a topology-controlling moiety here that does not compromise the size of the inner cavity. This contrasts with the first study on the backbone steric bulk-controlled formation of octahedra by Severin and coworkers where the overall size of the ligand backbone was increased so much that it reaches into the central cavity, as will be discussed later.^122,123^

**Fig. 7:**
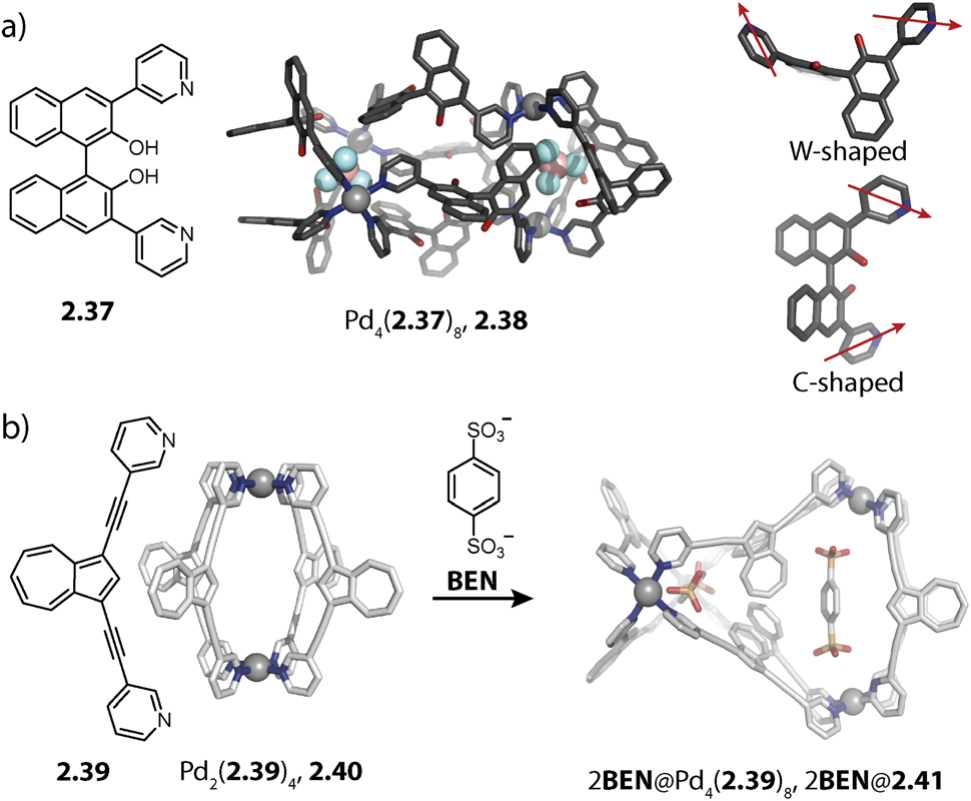
Templated self-assembly of the Pd_4_L_8_ distorted macrocycle and tetrahedron. (a) Chiral ligand 2.37 forms distorted tetrahedron 2.38 in which the ligands are present in a W-shaped and a C-shaped conformation. Two tetrafluoroborate counter anions are encapsulated in the outer pockets. (b) Azulene-based ligand 2.39 assembles to form lantern shaped cage 2.40 which shows guest-induced conversion to a distorted tetrahedron 2.41.

Lützen and coworkers applied chiral BINOL-based ligand 2.37 for assembling a tetranuclear species Pd_4_(2.37)_8_, 2.38 in which the Pd(ii) ions are arranged in a tetrahedral fashion (Fig. 7a). In contrast to the aforementioned tetrahedra possessing singly and doubly bridged edges, all neighboring metal ions are doubly bridged in 2.38. In this distorted macrocyclic structure, the ligands adopt two distinct conformations: ligands bridging the long edges have a W-shaped conformation with rather divergent binding angles while ligands bridging the short edges possess a C-shaped conformation with a smaller binding angle. This arrangement results in three pockets: two smaller peripheral pockets, each encapsulating one tetrafluoroborate anion, and a central cavity. Self-assembly of 2.37 with other Pd(ii) sources did not result in a defined species, showing that the tetrafluoroborate anions serve as the template for 2.38.^124^

We recently reported an unprecedented structural switch between a lantern-shaped cage Pd_2_(2.39)_4_, 2.40 and a distorted tetrahedron Pd_4_(2.39)_8_, 2.41 (Fig. 7b). Bis-pyridyl ligand 2.39 with an azulene backbone assembles with Pd(ii) to a classical lantern-shaped cage 2.40, as can be expected based on the geometry of the ligand (Chapter 2.1). Conversion into 2.41 was achieved upon addition of 1,4-benzene-bissulfonate (BEN). While the conformation of the ligands sitting on the doubly bridged edges closely resembles the situation in the lantern-shaped cage, the pyridines of the ligands on the singly bridged edges are flipped by approximately 180°. The shorter Pd–Pd distances, connecting the nodes of the double-bridged edges, match the size of the BEN guest, allowing for close attractive electrostatic interactions. Hence, maximizing host–guest interactions is presumably the driving force for the observed structural switch, aided by an energetic contribution resulting from a circular arrangement of the dipolar backbones of the singly bridged edges. Intriguingly, the switch could be reversed by addition of a competitive host.^79^

### 3.3. Hexanuclear species

**Fig. 8:**
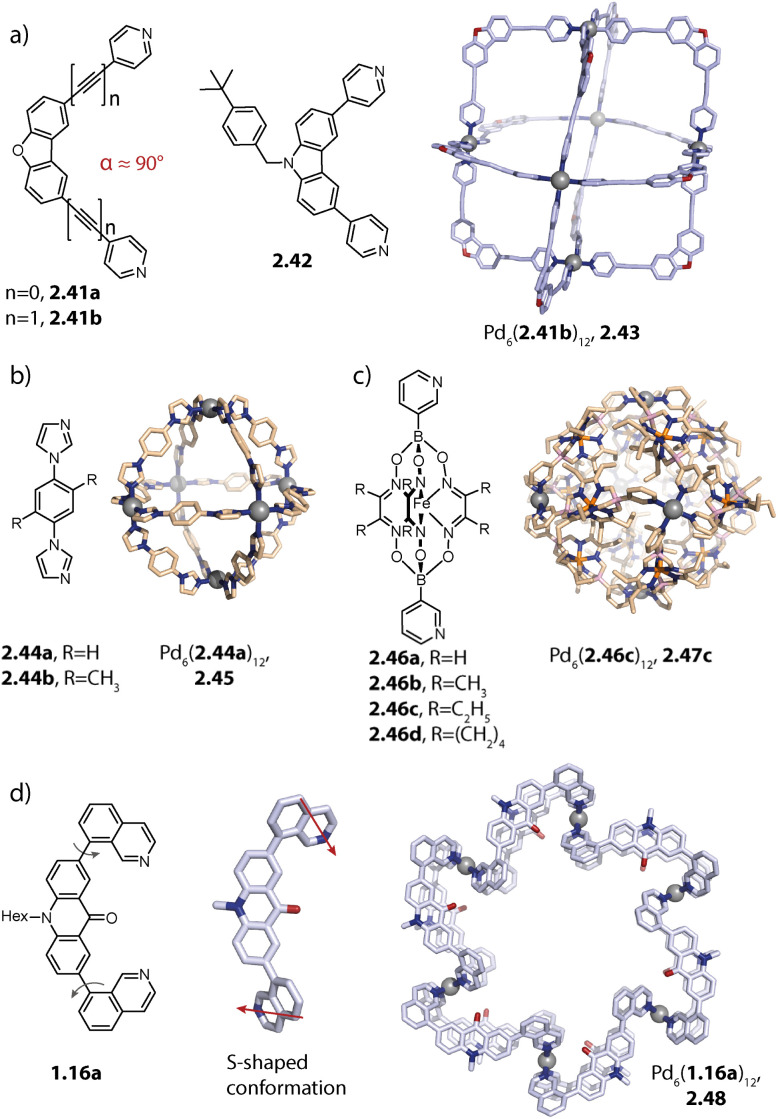
Homoleptic Pd_6_L_12_ assemblies. (a) Cubes (with an octahedral metal arrangement) based on rigid bis-*para* pyridyl ligands with binding angles close to 90°. (b) Octahedron 2.45 based on a short bis-imidazole terminated ligand. (c) Octahedron 2.47c based on bis-*meta* pyridyl ligands with a sterically crowded backbone. (d) Six-jagged ring 2.48 based on flexible ligand 1.16a adopting an S-shaped conformation.

Rigid ligands with nearly rectangular geometry assemble to form cubes in which the Pd(ii) ions are arranged in an octahedral fashion. This can be regarded as an extension of the above discussed series of rigid ligands to even larger binding angles. The first cube Pd_6_(2.41b)_12_, 2.43 was reported by Fujita and coworkers in 2009 and is assembled from dibenzofurane ligand 2.41b (Fig. 8a).^125^ Also, smaller ligand 2.41a and carbazole-based ligand 2.42 assemble to form analogous cubes, showcasing the reliability of the design approach based on the binding angle of rigid ligands. Reek and coworkers showed that cubes Pd_6_(2.41a)_12_ and Pd_6_(2.42)_12_ bind fullerene and that the binding affinity can be tuned by altering the electronic properties of the ligands.^126^

Furthermore, the group of Lützen reported BINOL-based ligands which possess, in contrast to ligand 2.37 discussed above, *para*-pyridine donor groups and hence larger binding angles. The ligands self-assemble to form Pd_6_L_12_ cubes and Pd_12_L_24_ spheres.^127^

Mukherjee and coworkers reported the first octahedra based on bis-imidazole ligands 2.44 (Fig. 8b). Similar to the cube-like structure, the Pd(ii) ions in Pd_6_(2.44)_12_, 2.45 are arranged in an octahedral fashion but due to the different ligand geometry, neighboring Pd(ii) are bridged in a rather linear fashion.^128^

Severin and coworkers reported clathrochelate ligands 2.46 which possess a similar geometry compared to ligand 2.24 introduced by the group of Fujita. However, the increased thickness of the backbone allowed for topology control: while less bulky 2.46a assembles to form the entropically favoured tetrahedron Pd_4_(2.46a)_8_, ligands 2.46b–d with increased steric bulk only allow the formation of larger octahedra Pd_6_(2.46b–d)_12_, 2.47b–d (Fig. 8c). Presumably, the larger species is favoured here in order to avoid repulsive steric interactions between the ligands in the smaller assembly. Furthermore, the authors showed with the help of enforced disassembly experiments that the decreased thickness of ligand 2.46d as compared to 2.46c translates into an enhanced thermodynamic stability of 2.47d. In turn, narcissistic self-sorting was observed when ligand 2.46d was combined with ligand 2.24. In contrast, a combination of 2.46c, 2.24, and Pd(ii) resulted in a library of mixed-ligand species.^122,123^

Recently, we reported the surprising conformational flexibility of short acridone-based ligand 1.16a. In solution, helically twisted Pd_2_(1.16a)_4_ is formed which is, depending on the solvent and counter anion, transformable into its mesocate isomer. Surprisingly, when we attempted to crystallize the mesocate, six-jagged ring Pd_6_(1.16a)_12_, 2.48 was obtained (Fig. 8d). Here, the ligands adopt an S-shaped conformation and pairs of ligands bridge neighbouring Pd(ii) ions, resulting in an overall *C*_6h_-symmetric structure. Crystal packing analysis revealed that the rings stack cofacially to form columnar, hexagonal channels that are separated by triangular gaps. The templated six-membered ring 2.22 reported by Sun and coworkers as described earlier and this solid-state structure are the first reported Pd_6_L_12_ rings.^94^

### 3.4. Interlocked and other unusual motifs

Lantern-shaped coordination cages can undergo dimerization through catenation. Thereby, the upper part of the ligands of one cage penetrates through the windows of the other cage. The most frequently observed interlocked double cages possess a fourfold symmetry and an alternating stack of Pd(ii) cations and counter anions which reduces the Coulomb repulsion between the closely positioned Pd(ii) ions. We have reviewed interpenetrated double cages in depth in the past^129^ and here, we will focus on some factors that allow for controlling interpenetration and on a recently reported new motif.

In 2008, Kuroda and coworkers reported Pd_4_L_8_ interlocked double cages for the first time. In this work, a combination of an organic ligand and Pd(NO_3_)_2_ in DMSO led to the formation of a monomeric cage Pd_2_L_4_ as a kinetic intermediate which was then converted into the quadruply interlocked double cage Pd_4_L_8_. Interestingly, when other Pd(ii) sources were used for the self-assembly, the equilibrium was shifted towards the monomeric cage. Also, the addition of a naphthalene-monosulfonate guest led to a preference for the monomeric cage.^130^

**Fig. 9:**
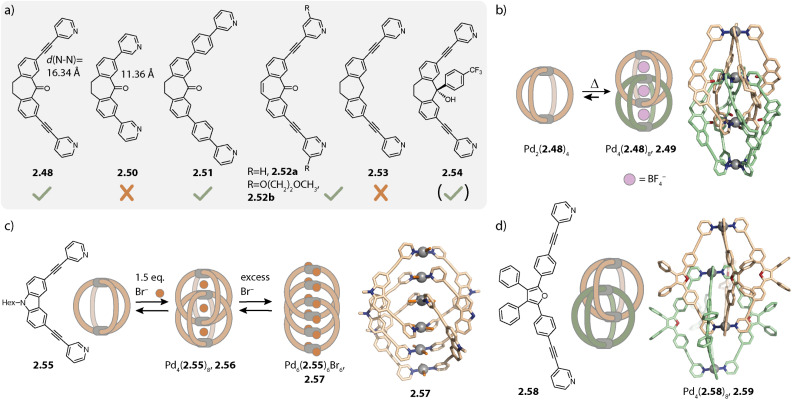
Interlocked double cages Pd_4_L_8_. (a) Structures of suberone-based ligands 2.48–2.54 (check mark: forms a double cage; cross: does not form a double cage). (b) Self-assembly of monomeric cage Pd_2_(2.48a)_4_ as a kinetic product and double cage 2.49a as the thermodynamic product. (c) Halide induced formation of double cage 2.56 and ligand release upon addition of excess halides to form triple catenated 2.57 in the solid state. (d) Triply interlocked double cage 2.59 with furane based ligand 2.58.

Later, we reported that dibenzosuberone-based ligand 2.48 assembles to form a similar interpenetrated cage Pd_4_(2.48)_8_, 2.49 with [Pd(CH_3_CN)_4_](BF_4_)_2_ in acetonitrile (Fig. 9b). Akin to the report of Kuroda and coworkers, the monomeric cage was observed to be a kinetic intermediate. In contrast to ligand 2.48 with alkyne spacers, double cage formation is not observed for ligand 2.50 with a shorter N⋯N distance (11.36 Å *vs.* 16.34 Å) under the same conditions. The pockets of a hypothetical double cage Pd_4_(2.50)_8_ would be too small for the incorporation of BF_4_^−^ (or any other) anions, resulting in a preference for the monomeric cage Pd_2_(2.50)_4_.^131^

For further controlling double- *vs.* monomeric cage formation, we investigated how structural modifications of the ligand 2.48 affect its ability to dimerize. Elongation of the linker by exchanging the alkyne with a phenyl spacer (2.51) and desaturation of the ligand backbone (2.52a) as well as modifications of the pyridines of 2.52 with methoxy ethoxy chains (2.52b) did not impede interpenetration. In contrast, deoxygenation of the backbone (2.53) led to formation of the monomeric cage Pd_2_(2.53)_4_. Close examination of the X-ray crystal structure in combination with DFT calculations revealed that the double cage structure 2.49 seems to be further stabilized by interactions between the oxygens of the carbonyl groups and the closely packed inner Pd(pyridine)_4_ plane of the other cage unit.^132^ This interaction, however, was not necessarily required for the interlocked cage reported by the group of Kuroda^130^ and for double cages with other ligands based on phenothiazine^133^ or helicene,^134^ reported by us.

The propensity for intercalation is also altered when inward-pointing steric bulk is installed on the ligand backbone. Derivative 2.54 which possesses the same N⋯N distance and binding angle as compared to 2.48 forms lantern-shaped cage Pd_2_(2.54)_4_ under similar self-assembly conditions to those used for obtaining double cage 2.49. However, the addition of chloride to Pd_2_(2.54)_4_ again resulted in double cage formation. This can be rationalized based on steric considerations: due to the comparably large size of BF_4_^−^ anions which would have to be encapsulated in the central pocket, the outer pockets of a potential cage BF_4_@Pd_4_(2.54)_8_ would be small, resulting in repulsive interactions between the attached aromatic groups. However, with chloride as the central anion, the outer pockets are enlarged, offering sufficient space for the bulky substituents.^135^ Yamashina, Toyota and coworkers showed, on the other hand, that exohedral steric bulk can also control dimerization.^136^

Similar chloride-induced interpenetration was observed with carbazole-based ligand 2.55 possessing shorter N⋯N distances as compared to 2.48. Only halides Cl^−^ and Br^−^, but not BF_4_^−^, can be sandwiched between the Pd(py)_4_ planes, allowing for catenation of two cages to give Pd_4_(2.55)_8_, 2.56 (Fig. 9c). In this case, addition of stoichiometric amounts of halides led, however, to a mixture of the interlocked double cage with the monomeric cage and the free ligand. Addition of over-stoichiometric amounts of halides leads to further ligand release. This can be explained by competition between halides and organic ligands coordinating to Pd(ii). Unexpectedly, triple catenated (PdBr_2_)_6_(2.55)_6_, 2.57, carrying no net charge, was crystallized. Here, the Pd(ii) ions are arranged in a linear stack with noticeably shorter Pd⋯Pd distances as compared to the distances in the corresponding double cage (4.36 Å *vs.* 6.73 Å). The bromide ligands are arranged in a helical fashion and the close proximity of the ligand backbones suggests favourable π–π interactions to be the key driving force for the observed structure.^137^

Bloch and coworkers reported the halide-triggered assembly of double cages that bind bisulfates with high affinity.^138^ The group of Hiraoka described a system of transformable quadruply interpenetrated cages in which different halide-dependent states lead to different reactivities.^139^

Most often, the degree of catenation is related to the symmetry of either the assembly or the ligand employed. This holds for the described quadruply interlocked cages with *C*_4_ or *D*_4_ symmetry, for triply interlocked *C*_3_ symmetric cages of Fujita,^140^ and for quintuply interlocked double cages based on *C*_5_ symmetric ligands reported by Nitschke and coworkers.^141^ However, recently, we reported unprecedented triply interlocked double cage Pd_4_(2.58)_8_, 2.59 with long furane-based ligand 2.58 (Fig. 9d). Similar to what has been reported for quadruply interlocked double cages, monomeric cage Pd_2_(2.58)_4_ was formed as a kinetic product. Heating led to a mixture of the monomeric cage with quadruply and triply interlocked double cage 2.59. At room temperature, the latter was observed to be the thermodynamic product. Here, two opposing windows of a first cage are each penetrated by one bridging ligand of a second cage while a third window of the first cage is penetrated by two ligands of the second cage. This leaves one window of the first cage free. The peculiar structure is stabilized by π–π interactions between the electron rich furane backbones and the electron poor pyridines. A reference ligand with the same geometry as 2.58 but a silole backbone that possesses additional methyl substituents assembles to form a monomeric cage. Due to the methyl substituents, π–π interactions involving the backbones are hampered, which highlights the necessity of intense π–π interactions for the formation of an interlocked cage.^142^

**Fig. 10:**
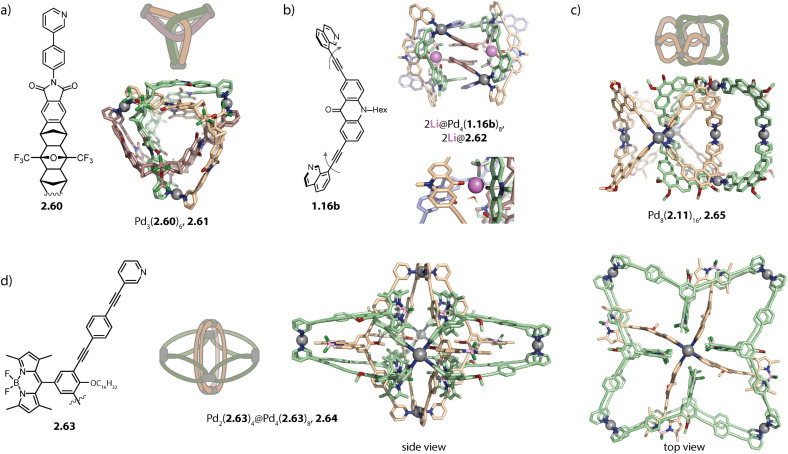
Interwoven and interpenetrated structures Pd_*n*_L_2*n*_, *n* = 3–8. (a) Double trefoil knot 2.61. (b) Li(i) templated low-symmetry assembly 2.62. (c) Octanuclear assembly 2.65 consisting of two four-membered rings 2.11 that are interlocked and rotationally displaced by 90°. (d) Pseudo-rotaxane cage-in-ring assembly 2.64 formed by BODIPY-functionalized ligand 2.63.

Aside from a simple three-membered ring, a double-trefoil knot structure Pd_3_(2.60)_6_, 2.61 was observed for the stoichiometry Pd_3_L_6_ when long concave ligand 2.60 was employed (Fig. 10a). Similar to the ring, each Pd(ii) pair is bridged by two organic ligands; however, the ligands are intertwined. This allows for close interactions between ligands 2.60 which possess multiple sites for π–π and H-bond interactions. Among the different possibilities for the arrangement of the ligands in 2.61, the peculiar structure was assigned employing a combination of NOESY NMR spectroscopy and molecular modelling, considering the symmetry of the ligands and the measured *vs.* calculated H⋯H distances. Double trefoil knot 2.61 consists of two chiral hemispheres with opposite configurations, forming an overall *meso* structure. The lack of preference for chiral guests of opposite configurations is thus in full accordance with the proposed structure.^143^

Severin and coworkers recently reported a structural switch from acridone based helicate Pd_2_(1.16b)_4_ to tetranuclear low symmetry assembly Pd_4_(1.16b)_8_, 2.62 upon addition of a Li salt. The structure consists of two Pd_2_(1.16b)_3_ distorted bowls that are bridged by two more ligands 1.16b which sit in the centre of the structure and are involved in π–π interactions (Fig. 10b). Two binding pockets are each occupied by one LiBF_4_ and a water molecule. The Li cation is stabilized by a tetrahedral environment of BF_4_^−^, water, and two carbonyl oxygens of the acridone ligands. The water molecule is furthermore engaged in H-bonds to two adjacent carbonyl oxygen atoms. Under strictly dry conditions, the peculiar, compact structure is not observed, underscoring the importance of the water molecules. Furthermore, the structural switch was selective for Li as it could not be induced by addition of other alkali metal salts.^144^ The same group incorporated lantern-shaped cages with polymerizable appendices as dynamic crosslinks in acrylamide hydrogels.^145^

Lützen and coworkers reported a cage-in-ring assembly Pd_2_(2.63)_4_@Pd_4_(2.63)_8_, 2.64, resembling a rotaxane (Fig. 10d). The shape of ligand 2.63 reminds to simple banana-shaped ligands known to assemble to form lantern-shaped cages Pd_2_L_4_. However, it stands out due to the length of its arms and possesses BODIPY and alkoxy substituents, having a propensity for engaging in π–π and van-der-Waals interactions, respectively. In the centrally positioned lantern-shaped cage Pd_2_(2.63)_4_, the ligands adopt a C-shaped conformation, leading to outward-pointing BODIPY-moieties and inward-pointing alkoxy chains. The ligands forming the four-membered ring Pd_4_(2.63)_8_ possess a W-shaped conformation in which the pyridine moieties are flipped by approximately 180°. Hence, the BODIPY substituents are endohedrally located allowing for π–π interaction with the backbones of Pd_2_(2.63)_4_. Additionally, the outer ring possesses clefts which enable the intercalation of the BODIPY moieties of Pd_2_(2.63)_4_.^146^

Catenation of Pd_*n*_L_2*n*_ architectures with *n* > 2 was so far only observed for four-membered rings 2.12 based on phenanthrene ligand 2.11. Octanuclear Pd_8_(2.11)_16_, 2.65 consists of two mechanically interlocked *D*_4h_-symmetric rings 2.12 that are rotationally displaced by 90° (Fig. 10c). The assembly displays *D*_2d_ symmetry and can be described as a huge Hopf-link. Noteworthily, the interlocked motif was only observed in the presence of nitrate, either upon direct assembly with Pd(NO_3_)_2_ or upon addition of a nitrate salt to a preformed mixture of tri- and tetranuclear assemblies. Presumably, nitrate anions possess the ideal size for templating the dimerization. Furthermore, switching the solvent from acetonitrile to DMSO impeded interlocking, similar to the dimerization of most lantern-shaped cages. Furthermore, higher order assembly to vesicular structures was achieved by equipping ligand 2.11 with hexyloxy chains. Each modified 2.65 assembly carries 32 hexyloxy chains, promoting aggregation *via* hydrophobic interactions in acetonitrile.^147^

To conclude, assemblies of higher nuclearity can be accessed with ligands possessing large bent angles, with flexible ligands and a suited template, or through interlocking of lower nuclearity assemblies. Starting off with architectures of rather high symmetry, namely rings, tetrahedra, and octahedra, we presented how the assembly size can be rationalized with the bent angle of the underlying ligand. We have also discussed more sophisticated architectures such as a cage-in-ring assembly, a double trefoil knot, and interlocked rings, among others, found serendipitously. Here, solvent and templation effects as well as ligand flexibility challenge the rationalization and prediction of the outcome.

## 4. Integrative self-sorting

As we can see, the investigation of homoleptic cage assembly has already produced an astonishing variety of topologies and it has enabled the rationalization of some key design principles. However, the functionality of such cages is inevitably limited as they carry only one kind of organic ligand – the building block that usually imbues the assembly with its function. For overcoming this constraint, multiple kinds of ligands can be combined, giving rise to so-called heteroleptic assemblies.^64,148^ The synthesis of such multicomponent cages is, however, not trivial in terms of the self-assembly strategy: when ligands of too similar size and shape are combined, the formation of an entropically favoured statistical mixture (*i.e.* assemblies with different stoichiometries and stereo configurations) is usually observed.^148,149^ The drawback of working with such mixtures is that only a subset of assemblies then comes with the desired functionality, and observed properties may not be correlated with a particular structure, impeding the optimization of the most selective and capable derivatives. For limiting the number of possible stoichiometries and isomers, *cis*-protected Pd(ii) sources have been successfully employed in the past. This has been combined with steric constraints by Fujita,^150^ with differently charged donors by Mukherjee and others,^151,152^ as well as with variation in the ligand denticity^153^ to achieve integrative self-sorting. Subsequently, strategies for the non-statistical self-assembly of bis-monodentate organic ligands carrying pyridine, isoquinoline, or imidazole donors with naked Pd(ii) ions were developed by Crowley,^154^ Hooley,^155^ Severin,^156^ and us.^80,99,121,157^ We will first describe Pd_2_L_4_ heteroleptic cages as they represent the most basic and largest family and will subsequently address heteroleptic cages of higher nuclearity.

### 4.1. Pd_2_A_2_B_2_ cages

**Fig. 11:**
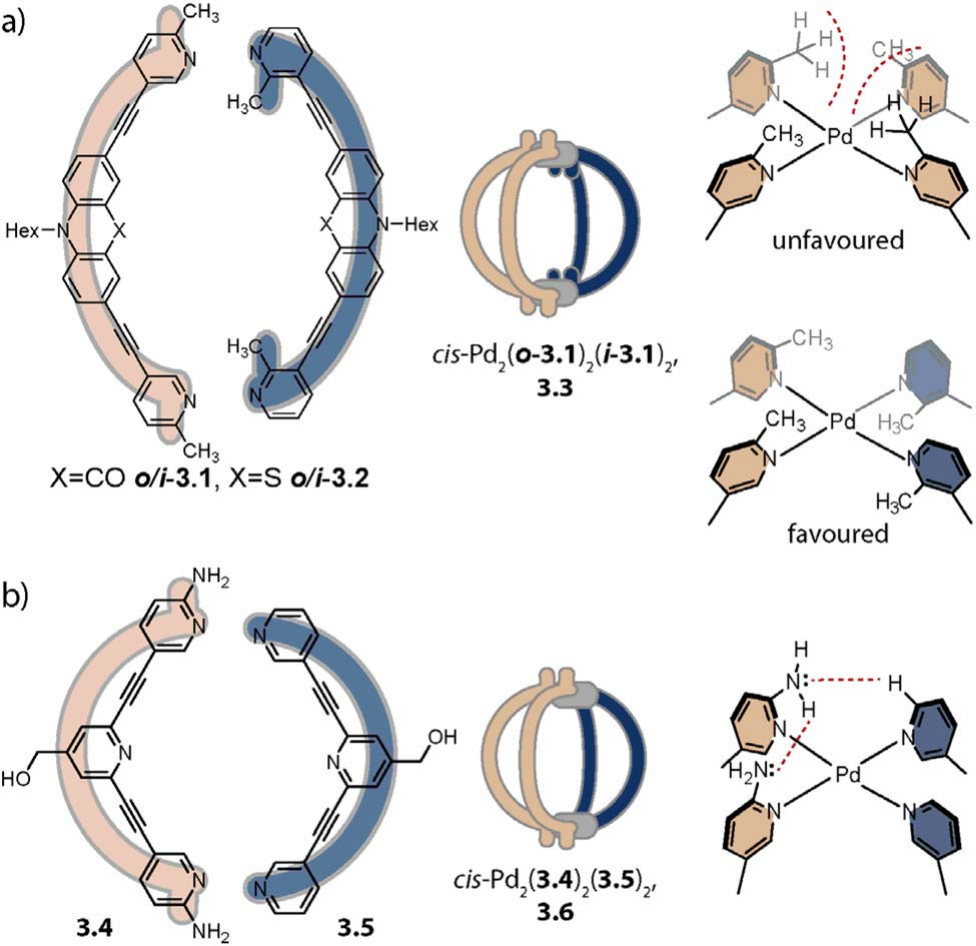
Integrative self-sorting to *cis*-Pd_2_A_2_B_2_-type cages using the CSE approach. (a) Repulsive steric interactions between the picolyl methyl groups destabilize a homoleptic with respect to a heteroleptic coordination environment resulting in 3.3 as the thermodynamic product. (b) Attractive interactions between the donor moieties kinetically stabilize cage 3.6.

Inspired by the work of Fujita and coworkers on heteroleptic assemblies based on *cis*-protected Pd(ii),^150^ we exploited steric constraints at the donor sites for yielding Pd_2_A_2_B_2_-type cages with naked Pd(ii) cations. For this, we coined the term “coordination sphere engineering” (CSE). Picolyl ligands *o*-3.1 and *i*-3.1 differ from the typical banana-shaped ligands discussed in Chapter 1 in the methyl-group adjacent to the nitrogen donor, either pointing outside (*o*-3.1) or inside (*i*-3.1) the tentative cavity of the respective assembly (Fig. 11a). Increased steric repulsion renders potential homoleptic species Pd_2_(*o*-3.1)_4_ or Pd_2_(*i*-3.1)_4_ energetically unfavourable; indeed, bowls or mixtures of bowls and rings are obtained when only one kind of ligand is employed. In contrast, the combination of both ligands *o*-3.1 and *i*-3.1 in a 1 : 1 ratio results in the formation of heteroleptic cage *cis*-Pd_2_(*o*-3.1)_2_(*i*-3.1)_2_, 3.3. Here, the repulsive steric interactions are decreased while the principle of maximum site occupancy is satisfied. DFT calculations and the X-ray crystal structure of a mononuclear Pd(picolyl)_4_ complex support that the *cis*-isomer possesses higher stability as compared to a tentative *trans*-isomer. The combination of acridone-based *o*-3.1 with phenothiazine-based *i*-3.2 led to a similar Pd_2_(*o*-3.1)_2_(*i*-3.2)_2_ cage, *i.e.* the platform tolerates incorporation of two different functional backbones. Surprisingly, the reverse combination of *i*-3.1 with *o*-3.2 did not allow for clean self-assembly to heteroleptic Pd_2_(*i*-3.1)_2_(*o*-3.2)_2_.^99^ This was ascribed to the high propensity of the phenothiazine-based ligand to form interlocked double cages.^133^ This study further revealed that this approach of sterically modifying the donor groups is not independent from the backbone geometries, in particular when the latter differs in the structure (with acridone being flat and phenothiazine being significantly bent along the tricyclic aromatic system) in that the degree of Pd(donor)_4_ helicity, as dictated by the methylated pyridines, will be mechanically linked to the backbone-based central geometry of such a cage.

Crowley and coworkers exploited attractive interactions around the coordination sites to steer integrative self-sorting. They designed ligands 3.4 and 3.5, differing solely in the presence or absence of amino groups on the pyridines. The combination of 3.4 with homoleptic cage Pd_23.54_ led to the formation of cage *cis*-Pd_2_(3.4)_2_(3.5)_2_, 3.6 for which the stereo configuration was assigned based on DFT computations (Fig. 11b). Preference for the *cis*-configuration was rationalized with the possibility for H-bond interactions between adjacent amino groups and additional H-bonds to the acidic α-protons of 3.5. In line with DFT computations that predict homoleptic Pd_2_(3.4)_4_ to be the most thermodynamically stable system, heteroleptic *cis*-Pd_2_(3.4)_2_(3.5)_2_ could not be accessed when the ligands, followed by the Pd(cations), or the preformed homoleptic cages were combined, *i.e.* it is a metastable, kinetic product.^154^

Aside from steric constraints, geometrical principles have also been exploited for the formation of heteroleptic assemblies. Examples of this include metallacycles with *cis*-protected Pd(ii) or Pt(ii) by Stang^158^ and Cu(ii)-based coordination cages by Zhou,^159^ both exploiting different ligand bent angles, and Pd_12_A_12_B_12_ spheres by Fujita whereby A and B possess different lengths.^160^

**Fig. 12:**
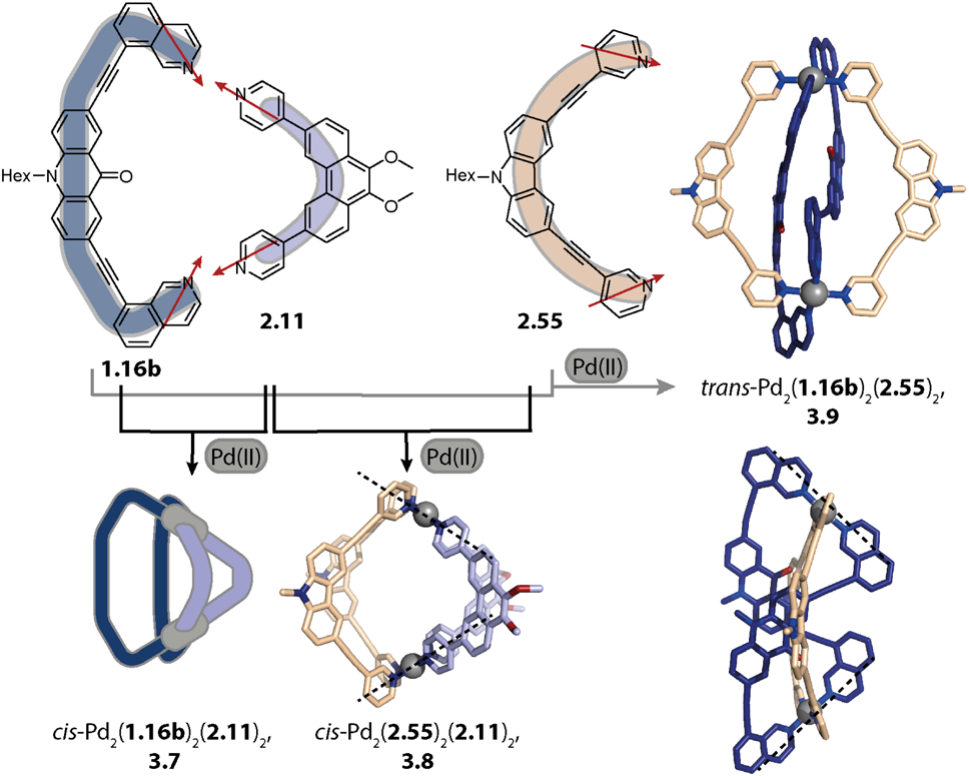
Integrative self-sorting to Pd_2_A_2_B_2_-type cages using the SCA approach. Shape complementarity between ligands 1.16b or 2.55 and 2.11 permits selective formation of 3.7 and 3.8, respectively. Combination of ligand 1.16b with 2.55 leads to *trans*-configurated 3.9. The donor vectors are highlighted with red arrows and the tilted Pd(ii) coordination planes with dashed lines.

Concerning Pd_*n*_L_2*n*_ cages, we introduced in 2016 a strategy exploiting complementary ligand geometries for achieving control over self-sorting and coined it “shape complementary assembly” (SCA).^80,103^ Ligands with convergent (1.16b or 2.55) and divergent (2.11) binding vectors are combined to yield a single heteroleptic assembly in a non-statistical fashion (Fig. 12). Matching binding angles and suitable lengths allow for a square-planar coordination environment of the Pd(ii) nodes, *e.g.* in *cis*-Pd_2_(1.16b)_2_(2.11)_2_, 3.7 and *cis*-Pd_2_(2.55)_2_(2.11)_2_, 3.8 without significant conformational strain. The severe twisting of the homoleptic helicate Pd_2_(1.16b)_4_ in combination with the high nuclearity of the homoleptic ring Pd_4_(2.11)_8_ drives the exergonic formation of 3.7, both from an enthalpic and an entropic point of view. Addition of ligand 1.16b to heteroleptic 3.8 results in transformation into 3.7 due to better shape complementarity of ligands 1.16b and 2.11. The complementarity of the binding angles leads to tilted Pd(donor)_4_ planes and therefore to a bent cavity shape. This translates into a higher guest binding affinity of 3.7 for bent as compared to linear disulfonate guests (opposite to a straight, homoleptic cage of comparable size). Unexpectedly, the combination of 1.16b and 2.55 also resulted in the formation of a defined species, *trans*-Pd_2_(1.16b)_2_(2.55)_2_, 3.9, in which ligands 1.16b adopt an S-shaped conformation and cross through the centre of ring Pd_2_(2.55)_2_ (Fig. 12; compare also to its homoleptic analogue in Fig. 3d). The SCA approach proved to be widely applicable and was exploited for the synthesis of amphiphilic cages,^161^ CPL-active cages,^162,163^ tetranuclear cages,^164^ and modular cage libraries for binding of phosphate esters.^165^

Recently, Jelfs Lewis and coworkers reported a joint theoretical and experimental approach aiming at predicting the integrative self-sorting of convergent and divergent ligands to give *cis*-Pd_2_A_2_B_2_ cages. From a theoretical perspective, the authors compared (a) the geometrical compatibility of ligand pairs and (b) the energy differences between homoleptic and heteroleptic species. The former yielded reasonable results in the sense that ligands for which integrative self-sorting was experimentally observed exhibited good geometrical compatibility. However, favourable geometric parameters did not ensure exclusive integrative self-sorting. Additionally, the relative energies of the overall assemblies were not congruent with the experimentally observed self-sorting outcome. These theoretical investigations were performed on single, static molecules, *i.e.* solvent and counter anion effects as well as entropic contributions were omitted in this simplified approach.^166^ In line with what was discussed for the topology control in homoleptic assemblies, these factors can be decisive for the assembly outcome, motivating to consider them in cage assembly rationalization or prediction schemes. Together with the Kast group, we recently showed that considering solvation effects gives valuable insight into homo-/heteroleptic assembly equilibria, while still being approximative and requiring further development.^167^

**Fig. 13:**
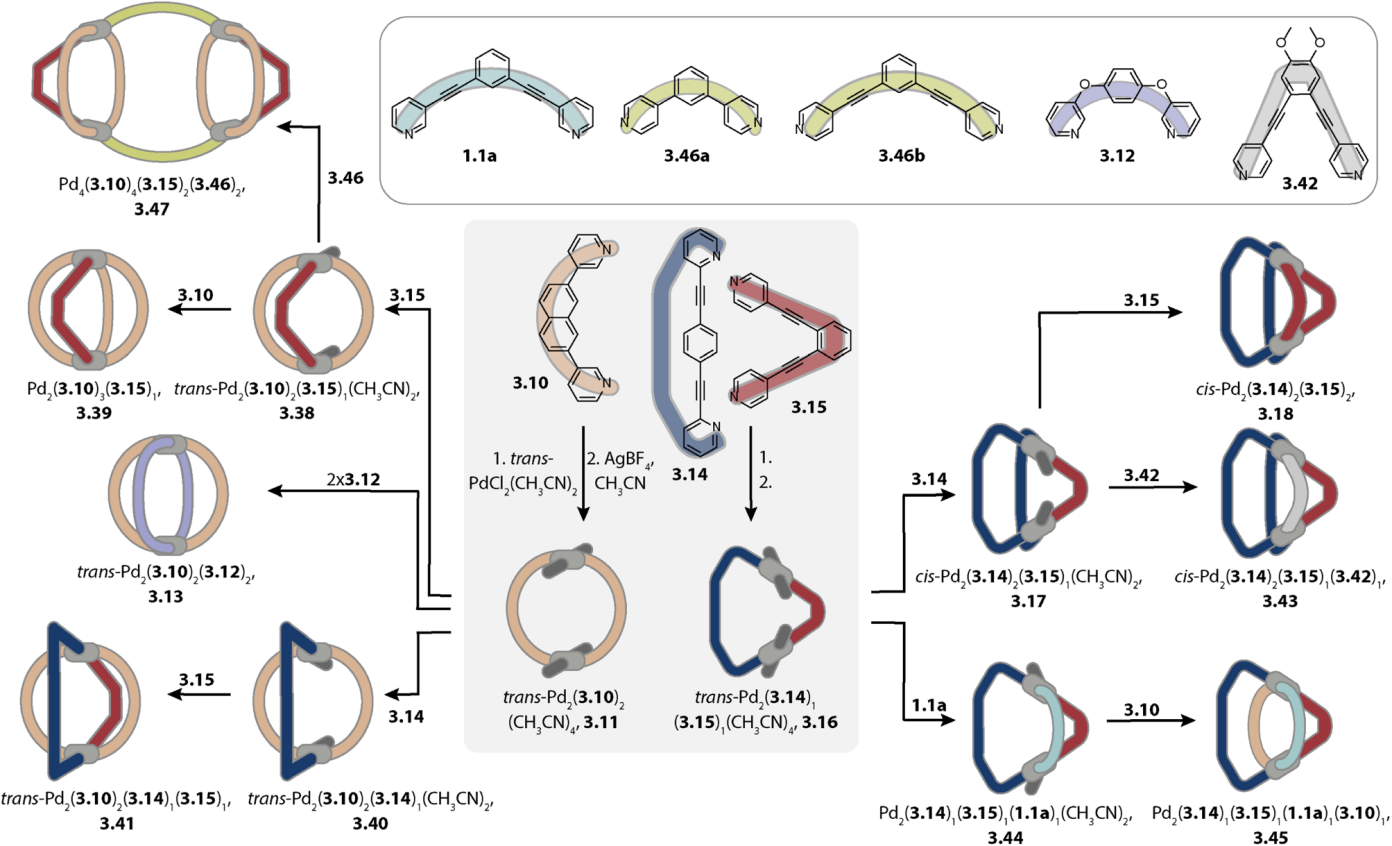
Synthesis of *cis*- and *trans*-Pd_2_A_2_B_2_ cages (3.18 and 3.13), Pd_2_A_3_B_1_ cage (3.39), *cis*- and *trans*-Pd_2_A_2_B_1_C_1_ cages (3.43 and 3.41), Pd_2_A_1_B_1_C_1_D_1_ cage (3.45), and tetranuclear Pd_4_A_4_B_2_C_2_ cage (3.47) under kinetic control.

Hiraoka and coworkers formed *cis*- and *trans*-Pd_2_A_2_B_2_ cages in a stepwise fashion under kinetic control. In the first step, the authors synthesized ring Pd_2_(3.10)_2_Cl_4_ (Fig. 13). Chloride abstraction in the presence of acetonitrile allows for the transformation into ring Pd_2_(3.10)_2_(CH_3_CN)_4_, 3.11 with kinetically labile acetonitrile ligands sitting in *trans*-position to each other. Addition of two equivalents of a further distinguishable ligand 3.12 allowed for the formation of *trans*-Pd_2_(3.10)_2_(12)_2_, 3.13. In a similar vein, ring *trans*-Pd_2_(3.14)_1_(3.15)_1_(CH_3_CN)_4_, 3.16 with shape complementary ligands 3.14 and 3.15 was synthesized. In a stepwise fashion, the acetonitrile ligands were replaced by an additional ligand 3.14 to form heteroleptic bowl Pd_2_(3.14)_2_(3.15)_1_(CH_3_CN)_2_, 3.17 followed by incorporation of ligand 3.15, yielding *cis*-Pd_2_(3.14)_2_(3.15)_2_, 3.18 within 4 steps in total. The key to successful kinetic control was (a) weaker donor strength of acetonitrile ligands as compared to pyridine ligands, (b) choice of solvent and counter anions that do not promote rapid ligand scrambling, (c) kinetic stability of the cyclic intermediates, and (d) local reversibility of ligand–metal bond formation due to the presence of the solvent acetonitrile, allowing for error correction.^168^

### 4.2. Pd_2_A_3_B_1_ cages

**Fig. 14:**
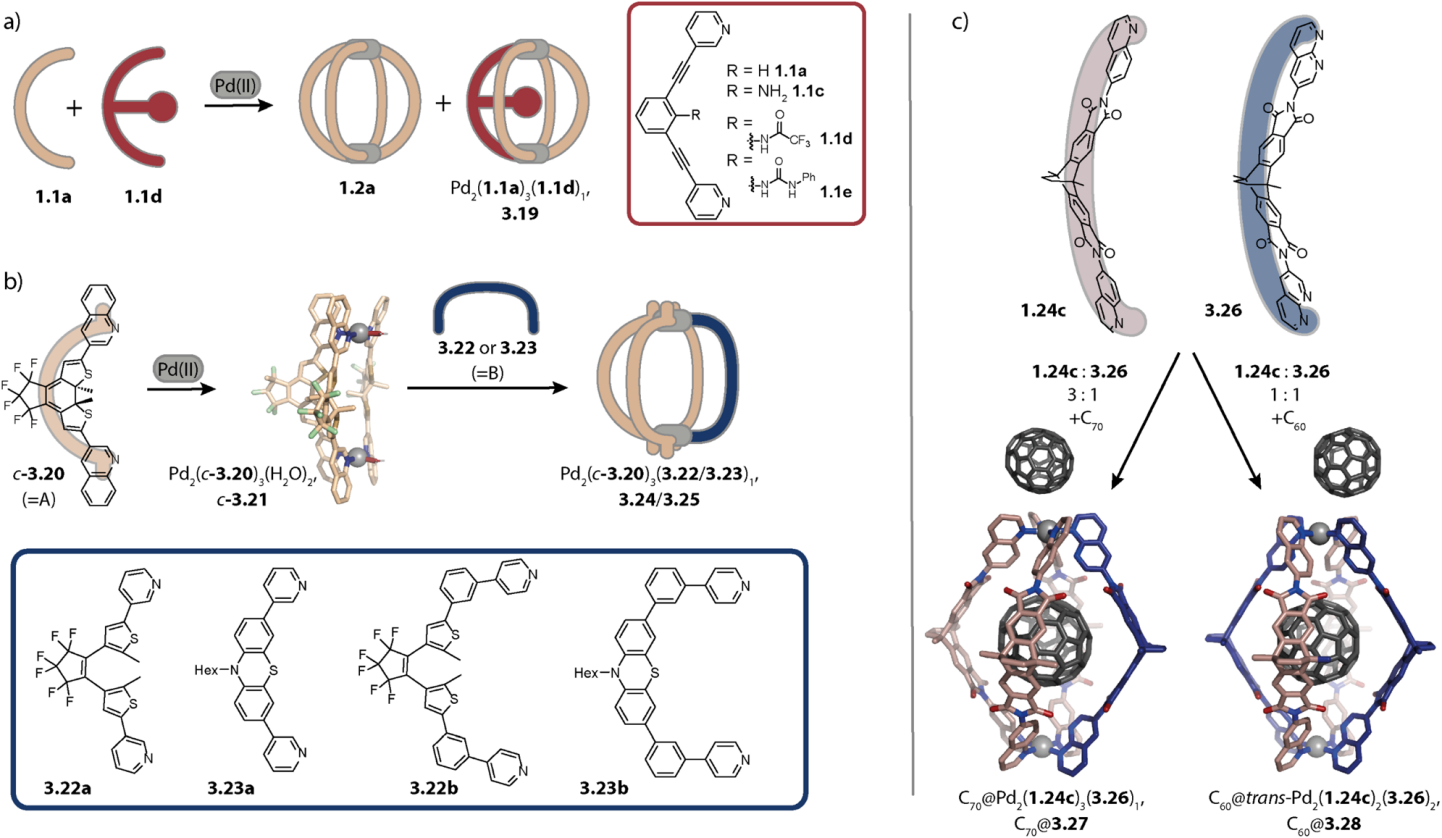
Strategies for the self-assembly of Pd_2_A_3_B-type cages. (a) Installation of endohedral bulk with a particular size allows for assembly to 3.19 along with the homoleptic cage 1.2a. (b) The CSE approach is applied for yielding bowl *c*-3.21 with “free” coordination sites that can be bridged by ligands 3.22a–3.23b lacking steric bulk at the donor moiety. (c) The CSE approach is expanded by attractive secondary interactions. Stoichiometry control then yields Pd_2_A_3_B_1_3.27 and *trans*-Pd_2_A_2_B_2_ cages 3.28 with encapsulated fullerenes.

Hooley and coworkers investigated in 2011 the effect of endohedral steric bulk on self-sorting. The authors synthesized derivatives of ligand 1.1a with substituents of increasing size on the central benzene ring (1.1c: amino group, 1.1d: trifluoroacetamide group, and 1.1e: *N*-phenyl urea, Fig. 14a). When ligand 1.1c was combined with 1.1a, a mixture of different species was obtained whose ratio could be varied by altering the ligand ratio. In contrast, the combination of 1.1a with 1.1d led to homoleptic 1.2a along with, for the first time, heteroleptic cage Pd_2_(1.1a)_3_(1.1d)_1_, 3.19. A further increase in steric bulk in 1.1e hampered the incorporation of the functionalized ligand into a lantern-shaped cage.^155^

The first selective self-sorting to Pd_2_A_3_B_1_ cages was achieved by us in 2021 utilizing the CSE approach. Photoswitchable DTE-based ligand 3.20 was equipped with quinoline donor moieties. Similar to quinoline ligands described earlier (Chapter 2.1), ligand *c*-3.20 (the DTE photoswitch is in its closed form) assembles to form homoleptic bowl Pd_2_(*c*-3.20)_3_(solvent)_2_, *c*-3.21 since repulsive steric interactions at the coordination site prohibit coordination of a fourth quinoline ligand (Fig. 14b). The introduction of a ligand of the same length, equipped with sterically less demanding pyridine donor moieties (ligands 3.22a–3.23b) allowed for occupation of the fourth coordination sites. This strategy proved robust across different fourth ligands and hence allowed for the synthesis of a series of cages Pd_2_(*c*-3.20)_3_(3.22a–3.23b)_1_, 3.24a–3.25b. Depending on the linker (no linker in 3.22a and 3.23a*vs.* a phenyl linker in 3.22b and 3.23b), the cages possess different cavity sizes which translates into size selective guest encapsulation.^169^

Moreover, we realized the installation of a fourth ligand on bowl-fullerene complex C_70_@1.25 in 2022. Again, coordination of a fourth ligand 1.24c with isoquinoline donor moieties is unfavoured due to steric repulsion at the coordination site. In contrast, when a similar ligand with naphthyridine donors 3.26 is employed, C_70_@Pd_2_(1.24c)_3_(3.26)_1_, C_70_@3.27 is obtained (Fig. 14c, left). In addition to the reduced steric bulk of the naphthyridine in comparison to isoquinoline, the nitrogen lone pairs of the former allow for attractive interactions with the hydrogens of the adjacent donor groups of 1.24c. Importantly, the CSE approach alone was not sufficient here for the clean formation of 3.27 in the absence of fullerene C_70_ as a template. Additionally, ligands 1.24c and 3.26 were combined in a 1 : 1 ratio with fullerene C_60_ as a template, whereby C_60_@*trans*-Pd_2_(1.24c)_2_(3.26)_2_, C_60_@3.28 was obtained (Fig. 14c, right). The combination of the CSE approach and templation by fullerene also allowed for the assembly of heteroleptic bowls: when acridine-equipped ligand 1.24d forming Pd_2_(1.24d)_2_(CH_3_CN)_4_ rings was combined with naphthyridine-equipped ligand 3.26 in the presence of C_60_ or C_70_, host–guest complexes C_60_/C_70_@*trans*-Pd_2_(1.24d)_2_(3.26)_1_(CH_3_CN)_2_ were selectively obtained. Here, naphthyridyl ligand 3.26 bridges one side of the ring Pd_2_(1.24d)_2_.^157^

**Fig. 15:**
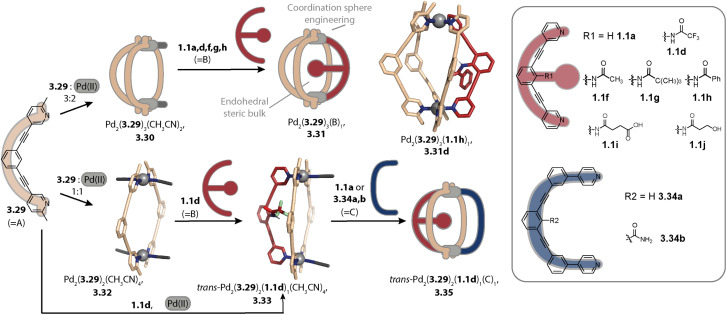
Synthesis of Pd_2_A_3_B_1_ (top) and Pd_2_A_2_B_1_C_1_ cages (bottom) through the incorporation of two steric control elements located at the donor moieties (CSE approach) and within the cage cavity. Picolyl ligand 3.29 assembles to form (metastable) bowl 3.30 or ring 3.32 depending on the ligand-to-metal stoichiometry. Bowl 3.30 can be converted into cage 3.31 with a fourth ligand lacking bulk at the donor site. The combination of ring 3.32 with a ligand equipped with endohedral bulk (shown for 1.1d) or the ligands with Pd(ii) yields heteroleptic bowl 3.33 which can be converted into Pd_2_A_2_B_1_C_1_-type cage 3.35.

In 2024, Zhang and coworkers combined the CSE approach with the incorporation of endohedral steric bulk to form Pd_2_A_3_B_1_ cages with varying cavity volumes. Picolyl ligand 3.29 was combined with Pd(ii) in a 3 : 2 ratio for obtaining metastable bowl Pd_2_(3.29)_3_(CH_3_CN)_2_, 3.30 (Fig. 15, top). In the next step, pyridine ligands 1.1a, 1.1d, 1.1f, 1.1g, and 1.1h were added as ligand B to form cages Pd_2_(3.29)_3_B_1_, 3.31. While this stepwise synthesis allowed for obtaining the peculiar heteroleptic cages with sufficient selectivity, a one-pot reaction of the ligands with Pd(ii) led to a mixture of different assemblies. For ligand B, possessing endohedral bulk (1.1d, 1.1f, 1.1g, and 1.1h), the heteroleptic cage 3.31 was, however, the main species. This showcases how the interplay of two steric control elements can steer integrative self-sorting. Noteworthily, the accessible cavity volume decreases depending on ligand B in the order 1.1a > 1.1f > 1.1g. This was shown to result in an alteration of the host–guest properties.^170^ Wang and coworkers also combined coordination sphere engineering and endohedral steric bulk for realizing Pd_2_A_2_B_2_ and Pd_2_A_3_B cages in a pathway-dependent approach.^171^

### 4.3. Pd_2_A_2_B_1_C_1_ cages

Zhang and coworkers reported in 2023 the first example of a heteroleptic Pd_2_A_2_B_1_C_1_-type cage incorporating three distinguishable ligands. Similar to their strategy for the synthesis of Pd_2_A_3_B_1_-type cages, the CSE approach was merged with the installation of endohedral steric bulk. The combination of picolyl ligand 3.29 with Pd(ii) in a 1 : 1 ratio led to ring Pd_2_(3.29)_2_(CH_3_CN)_4_, 3.32 (Fig. 15, bottom). Next, one side of the ring was bridged by ligand 1.1d, acting as a ligand “B”. While ligand 1.1d is of similar shape to 1.1a it possesses endohedral steric bulk, hampering coordination of a second equivalent. Thus, a heteroleptic bowl *trans*-Pd_2_(3.29)_2_(1.1d)_1_(CH_3_CN)_2_, 3.33 is obtained. Noteworthily, bowl 3.33 can also be assembled directly from a mixture of ligands 3.29 and 1.1d with Pd(ii), showing that its formation is pathway-independent. Lastly, a sterically less demanding ligand, such as unsubstituted 1.1a, can be implemented as ligand “C” to yield *trans*-Pd_2_(3.29)_2_(1.1d)_1_(1.1a)_1_, 3.35. The authors showed that different endohedral functionalities can be incorporated into the heteroleptic cage by changing the appended group in ligand B (1.1i: carboxylic acid and 1.1j: hydroxy group). Furthermore, the cavity can be enlarged by employing larger 3.34a as ligand C. On the other hand, a second endohedral moiety can be incorporated by functionalization of ligand C (3.34b).^172^

**Fig. 16:**
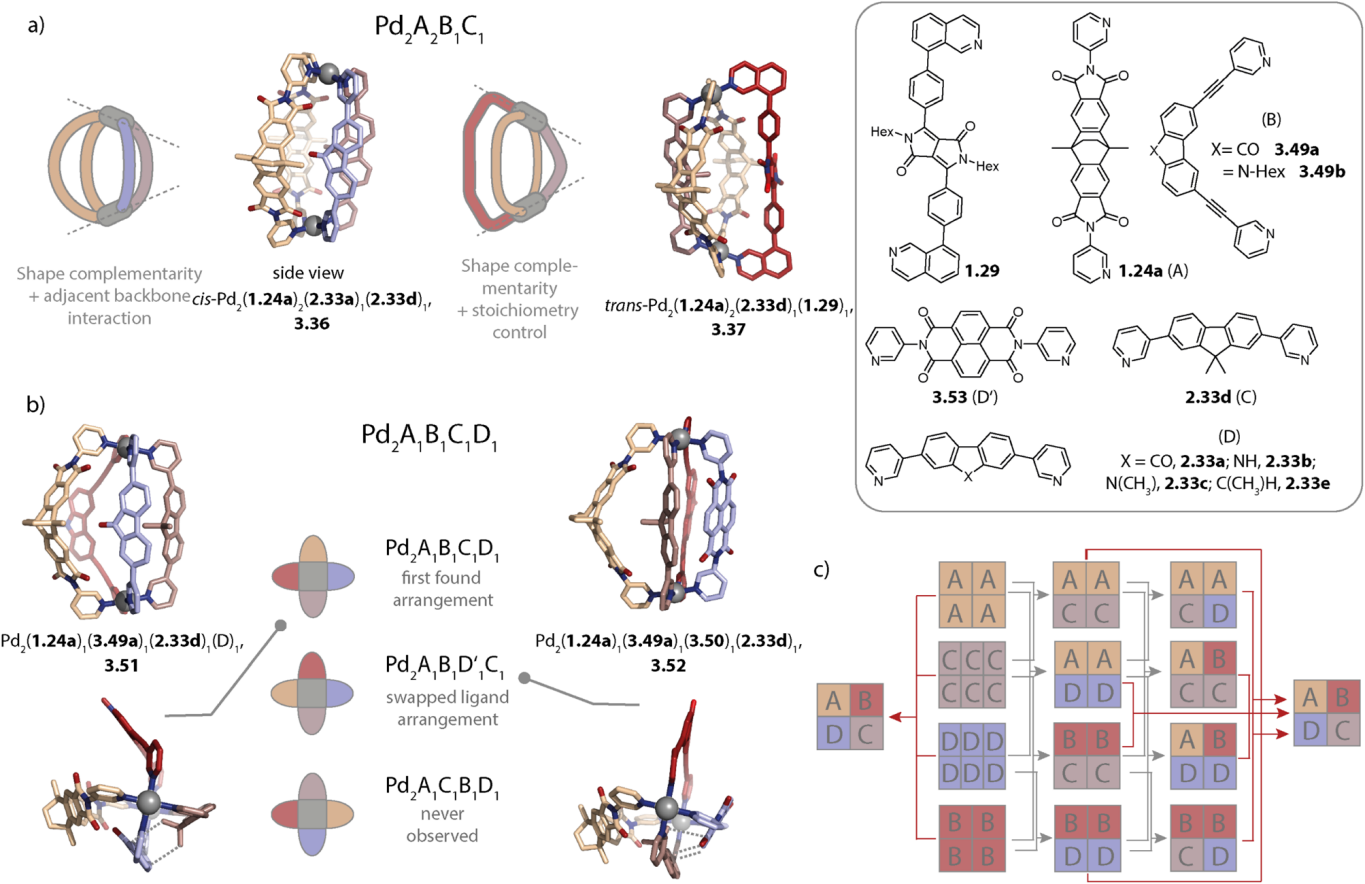
Integrative self-sorting to Pd_2_A_2_B_1_C_1_-type and Pd_2_A_1_B_1_C_1_D_1_-type cages under thermodynamic control. (a) Structures of *cis*-Pd_2_A_2_B_1_C_1_-type cage 3.36 and *trans*-Pd_2_A_2_B_1_C_1_-type cage 3.37 obtained by combining the SCA approach with the ABI approach or stoichiometry control, respectively. (b) Illustration of the three possible ligand arrangements in Pd_2_A_1_B_1_C_1_D_1_-type cages and X-ray structures of realized pseudo-isomers 3.51 and 3.52, selectively formed as one of 55 homo- and heteroleptic dinuclear cages possible in principle. (c) All possible pathways for the synthesis of the Pd_2_A_1_B_1_C_1_D_1_-type cage *via* cage-to-cage transformations.

In 2024, we combined the SCA approach with either inter-ligand interactions or with stoichiometry control to steer the selective formation of *cis*- or *trans*-Pd_2_A_2_B_1_C_1_ cages, respectively. In accordance with the SCA approach, ligand 1.24a assembles with ligands 2.33a or 2.33d to form *cis*-Pd_2_(1.24a)_2_(2.33)_2_ cages. While ligands 2.33a and 2.33d feature similar geometries, their electronic properties diverge: fluorene-based ligand 2.33d possesses two CH_3_ groups while fluorenone-based ligand 2.33a has a planar π-surface. CH_3_⋯π interactions between central parts of neighbouring ligands 2.33a and 2.33d foster selective formation of *cis*-Pd_2_(1.24a)_2_(2.33a)_1_(2.33d)_1_, 3.36 when the ligands are combined in a ratio of 2 : 1 : 1 with Pd(ii) (Fig. 16a). For this, we coined the term “adjacent backbone interactions” (ABIs). Small ligands 2.33 also show shape complementarity to ligand 1.29 with strongly inward pointing donor vectors. Therefore, when 1.29, 2.33d, and 1.24a are combined with Pd(ii) in a 1 : 1 : 2 : 2 ratio, *trans*-Pd_2_(1.24a)_2_(1.29)_1_(2.33d)_1_, 3.37 is obtained. That ligand 1.24a can adapt both, a conformation with rather convergent binding vectors, allowing for shape complementarity to ligands 2.33, and a conformation with nearly collinear binding vectors without considerable strain can be explained with its spring-like nature that enables adaptable lengths and binding angles.^121,173^

### 4.4. Pd_2_A_1_B_1_C_1_D_1_ cages

Utilizing the pathway-dependent self-assembly approach described earlier, Hiraoka and coworkers formed Pd_2_A_3_B_1_, PdA_2_B_1_C_1_, as well as the first Pd_2_A_1_B_1_C_1_D_1_-type cage under kinetic control. Ring 3.11 was reacted with ligand 3.15 to yield intermediate bowl Pd_2_(3.10)_2_(3.15)_1_(CH_3_CN)_2_, 3.38 that was then converted into Pd_2_(3.10)_3_(3.15)_1_, 3.39 by the addition of a further equivalent of ligand 3.10 (Fig. 13). Reversing the last two steps did not lead to selective formation of 3.39 since homoleptic Pd_2_(3.10)_4_ can be formed. When intermediate bowl 3.38 was reacted with ligand 3.14, which is complementary in shape to ligand 3.15, cage *trans*-Pd_2_(3.10)_2_(3.14)_1_(3.15)_1_, 3.41 was obtained. 3.41 was also obtained when ligands 3.14 and 3.15 were added in the reverse order. Notably, this cage exhibited thermodynamic stability. This can be explained similar to the stability of cage 3.37: the ligands opposing each other either possess parallel binding vectors (ligand 3.10) or complementary geometries (3.14 and 3.15). In another vein, intermediate bowl 3.17 yielded cage *cis*-Pd_2_(3.14)_2_(3.15)_1_(3.42)_1_, 3.43 when ligand 3.42, only differing from ligand 3.15 by its exohedral substituents, was added. The culmination of the synthetic strategy represents the formation of a cage with four differentiable ligands: starting from ring 3.16, bowl intermediate Pd_2_(3.14)_1_(3.15)_1_(1.1a)_1_(CH_3_CN)_2_, 3.44 was obtained upon addition of ligand 1.1a. With ligand 3.10, bowl 3.44 was then converted into Pd_2_(3.14)_1_(3.15)_1_(1.1a)_1_(3.10)_1_, 3.45. Except for cage 3.41, heating of all assemblies discussed here resulted in a transformation into other, thermodynamically more stable compounds. In order to get insight into the factors influencing the respective cage stabilities, the authors determined the half-life of the cages and correlated them with different structural features. It was found that small deviations of the N⋯N distances of the ligands in the cages from their ideal distances are essential for high cage stability.^168^

Finally, we published the first report on the thermodynamically controlled formation of Pd_2_A_1_B_1_C_1_D_1_-type cages in 2024. Based on our knowledge on the shape complementarity of larger ligands 1.24a (A) and 3.49 (B) with smaller ligands 2.33d (C) and 2.33a (D) as well as favourable inter-ligand interactions between the latter (ABI approach), we combined all four ligands with Pd(ii) cations in a 1 : 1 : 1 : 1 : 2 ratio. Intriguingly, out of 55 possible dinuclear species, one single assembly incorporating all four ligands was exclusively obtained, both in solution and the solid state. Considering the above-mentioned driving forces, two different isomers, namely Pd_2_(1.24a)_1_(3.49a)_1_(2.33d)_1_(2.33a)_1_, 3.51 (Pd_2_A_1_B_1_C_1_D_1_) and Pd_2_(1.24a)_1_(3.49a)_1_(2.33a)_1_(2.33d)_1_ (Pd_2_A_1_B_1_D_1_C_1_) would have been feasible whereby X-ray structure analysis revealed that only the former is formed (Fig. 16b). Concerning the observed stoichiometry, presumably, the incorporation of one equivalent of ligand 1.24a is favoured due to its ability to adapt its N⋯N distance to match the other ligands. Fluorenone-based ligand 3.49a could be replaced by carbazole-based ligand 3.49b, possessing a similar shape and flexibility. Furthermore, we showed that ligands 2.33b, 2.33c, and 2.33e, based on carbazole or fluorene, can likewise serve as ligand D since they also offer a π-surface for adjacent backbone interactions with ligand 2.33d. In contrast, dimethyl fluorene ligand 2.33d C proved to be vital for the selective outcome since substitution by ligands 2.33b, 2.33c, or 2.33e which offer only up to one CH_3_ group in the central backbone position resulted in the formation of mixtures of different species. Interestingly, when NDI-based ligand 3.53 was applied as ligand D, a swapped ligand arrangement was observed. In this case, ligand 2.33d offers its π-surface for CH⋯π-interactions with ligand 3.53. Probably, the alteration of the orientations of ligand C and D affects the shape complementarity with ligands A and B in a way that now favours the formation of pseudo-isomeric cage 3.52. Noteworthily, cage Pd_2_A_1_B_1_C_1_D_1_ could also be obtained in a stepwise fashion through several possible cage-to-cage transformation pathways, starting from the respective homoleptic cages, again supporting its characteristics as the final thermodynamic assembly product (Fig. 16c).^121^

### 4.5. Pd_*n*_A_*x*_B_*y*_ assemblies with *n* > 2

**Fig. 17:**
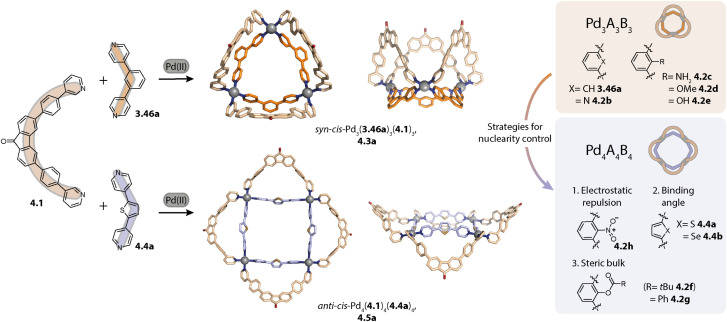
Self-assembly of Pd_3_A_3_B_3_-type and Pd_4_A_4_B_4_-type heteroleptic rings. (Left) Ligand 4.1 self-assembles with ligand 3.46a to form bowl-shaped 4.3a (top) and with ligand 4.4a to form saddle-shaped 4.5a (bottom). (Right) The ring nuclearity can be increased upon modification of the smaller ligand either through (1) the incorporation of a nitro substituent, (2) an increase in the bent angle, or (3) installation of steric bulk.

Recently, we reported the first trinuclear heteroleptic assemblies Pd_3_A_3_B_3_. By applying the SCA approach, the long, convergent ligand 4.1 was combined with the small, divergent ligand 3.46a. This resulted in the formation of three-fold symmetric *syn-cis*-Pd_3_(3.46a)_3_(4.1)_3_, 4.3a assembly (Fig. 17). Here, *cis* refers to the ligand arrangement around the metal node and *syn* to the ligand orientation relative to the Pd_3_ plane, *i.e.* the assembly is overall bowl-shaped. Modification of the central benzene moiety (ligands 4.2b–e) allowed for obtaining endohedrally functionalized rings 4.3b–e. Noteworthily, when uncharged and small functionalities were incorporated, the self-assembly to Pd_3_A_3_B_3_-type rings was not disturbed. On the other hand, when the sterically more demanding *tert*-butyl ester modified ligand 4.2f was employed, a mixture of trinuclear and tetranuclear assemblies was obtained. A further increase in steric bulk (phenyl ester ligand 4.2g) led to the tetranuclear ring as the sole species. In a similar fashion, functionalization with a nitro group led to higher nuclearity ring Pd_4_(4.1)_4_(4.2h)_4_, 4.5h. Presumably, this can be traced back to reduced electrostatic repulsion between the nitro oxygens of ligands 4.2h when occupying neighboring edges in the larger assembly as compared to a tentative three-membered ring. Aside from the incorporation of steric bulk and charges, the formation of a Pd_4_A_4_B_4_-type assembly could also be steered by modification of the bent angle of the small ligand. When the central benzene moiety in 3.46a (*α* = 120°) was substituted by a thiophene or a selenophene moiety, the bent angle was increased to 149° (4.4a) and 151° (4.4b), respectively. In accordance with the trends observed for homoleptic assemblies (Fig. 4), this led to an increase in nuclearity, here to a Pd_4_A_4_B_4_-type ring. Interestingly, NMR analyses in combination with DFT computations suggest that for most of the four-membered rings investigated, more precisely the rings formed with 4.4a, 4.4b, and 4.2g, a saddle-shaped conformation is favoured over a bowl-shaped one.^41^

**Fig. 18:**
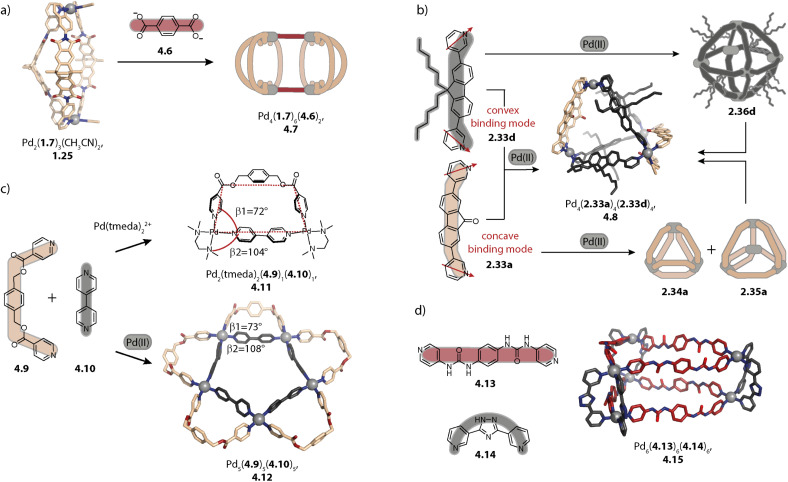
(a) Pill-shaped dimer 4.7 is obtained by bridging two counts of bowl 1.25 with dicarboxylate ligand 4.6. (b) Fluorenone-derived ligands 2.33a/d self-assemble either to form a mixture of ring 2.34 and tetrahedron 2.35 or to form an octahedron 2.36 depending on the backbone-centred steric bulk. Combination of 2.33a and 2.33d with Pd(ii) results in integrative self-sorting to heteroleptic tetrahedron 4.8. (c) Self-assembly of flexible ligand 4.9 with linear ligand 4.10 gives rise to trapezium-shaped complex 4.11 when a *cis*-protected Pd(ii) source is employed and to five-membered ring 4.12 with unprotected Pd(ii) (*β*_1_ and *β*_2_ are exterior and interior angles of the truncated pentagram). (d) Trigonal prismatic assembly 4.15 formed with urea-functionalized ligand 4.13 and ligand 4.14.

In 2019, we reported the self-assembly of pill-shaped tetranuclear species by exploiting the charge separation strategy, widely utilized by Stang and coworkers,^15^ combined with our CSE approach. As discussed in the preceding section, the “free” coordination sites of quinoline-based Pd_2_A_3_-type bowls (such as 1.25, Fig. 18a) can be occupied by ligands lacking steric bulk close to the donor site. When instead of a convergent bis-pyridyl ligand a linear terephthalate 4.6 is employed, two bowls are bridged, giving rise to pill-shaped Pd_4_(1.7)_6_(4.6)_2_, 4.7. The negative charges of the terephthalates help compensate for the repulsion stemming from the close proximity of multiple Pd(ii) cations. Additionally, the dimer-fullerene complexes 2C_60_@4.7 or 2C_70_@4.7 could also be accessed upon dimerization of preformed bowl-fullerene complexes C_60_@1.25 and C_70_@1.25.^76^

In another vein, Hiraoka and coworkers recently reported pill-shaped Pd_4_A_4_B_2_C_2_-type assemblies *via* dimerization of two Pd_2_A_2_B heteroleptic bowls under kinetic control. Starting from bowl 3.38, whose synthesis is described in Chapter 4.4, the authors added long or short bis-pyridyl ligands 3.46 with diverging binding angles. Similar to our approach, pill-shaped dimer Pd_4_(3.10)_4_(3.15)_2_(3.46)_2_, 3.47 was obtained, albeit here with three different bis-pyridyl ligands and higher positive charge (Fig. 13).^168^

In 2021, we reported a strategy for yielding heteroleptic tetrahedra by exploiting the role of backbone-centred steric bulk (as a special variant of the ABI approach). As described in Chapter 3.2, fluorene-derived ligands 2.33 can assemble to form rings, pseudo-tetrahedra, and octahedra, depending on the steric bulk installed at the backbone. While ligand 2.33a with low steric demand assembles to form a mixture of ring 2.34a and tetrahedron 2.35a, dihexyl-decorated ligand 2.33d forms entropically disfavoured homoleptic octahedron 2.36d (Fig. 18b). In the latter, the distance between the ligands is increased and they adopt a concave binding mode while the steric bulk is oriented to the exterior. Upon careful inspection of the ligand conformations and arrangement in tetrahedron 2.35a (Fig. 6), we suspected that the assembly could serve as a platform for combining sterically demanding ligands, sitting on the singly bridged edges, with ligands of low steric demand occupying the doubly bridged edges. Indeed, a combination of either ligands 2.33a and 2.33d with Pd(ii) or combination of the preformed homoleptic assemblies 2.34a and 2.35a with 2.36d resulted in the selective formation of tetrahedron Pd_4_(2.33a)_4_(2.33d)_4_, 4.8 with the expected ligand arrangement (Fig. 18b).^119^

Yoshizawa, Chand, and coworkers reported the first and, until now, sole pentanuclear heteroleptic assembly in 2017 (elected as the molecule of the year in ACS Chemical & Engineering News). In the first step, the authors combined the rather flexible ligand 4.9 with linear ligand 4.10 and a *cis*-protected Pd(ii) source, (tmeda)Pd(ii). A trapezium-shaped complex Pd_2_(tmeda)_2_(4.9)_1_(4.10)_1_, 4.11 with an exterior angle *β*_1_ of 72° and an interior angle *β*_2_ of 104° was obtained, serving as a promising precursor to a higher order assembly (Fig. 18c).^174^ It was then anticipated that the combination of the same two ligands with unprotected Pd(ii) should result in a circular arrangement of fused trapezium-shaped complexes 4.11. Comparing the angles observed in 4.11 with the angles in tentative triangular, square, and pentagonal arrangements of annelated trapezia suggested that, from a purely geometrical point of view, the pentagonal species should be favoured. Indeed, the combination of 4.9 and 4.10 with [Pd(CH_3_CN)_4_](BF_4_)_2_ in DMSO resulted in pentanuclear species Pd_5_(4.9)_5_(4.10)_5_, 4.12 which can be described as a truncated pentagram. Here, the interior angle reaches 108°, similar to the one of a regular pentagon. Noteworthily, ring 4.12 could also be accessed *via* different pathways, more precisely upon combination of the two homoleptic species or upon addition of one of the ligands to the homoleptic assembly of the respective other ligand, supporting that 4.12 is the thermodynamic product.^175^

In 2016, Mukherjee and coworkers reported a trigonal prismatic Pd_6_A_6_B_6_-type cage. First, the authors showed that urea-functionalized bis-pyridyl ligand 4.13 assembles with *cis*-protected (tmeda)Pd(ii) to form a molecular triangle Pd_3_(tmeda)_3_(4.13)_3_. Aiming at a three-dimensional assembly, ligand 4.13 was combined with unprotected Pd(ii) and short “clipping” ligand 4.14. In the resulting assembly Pd_6_(4.13)_6_(4.14)_6_, 4.15, in total six clipping ligands 4.14 bridge two triangles Pd_3_(4.13)_3_ (Fig. 18d). Urea-functionalized compounds are generally prone to self-association *via* hydrogen bonds which limits their capability of substrate recognition and hence their catalytic applicability. The large inter-ligand distances in the triangular prism prohibit such self-quenching interactions, rendering the assembly suitable for the recognition of nitro-olefins. Therefore, triangular prism 4.15 could be used as a heterogeneous catalyst for promoting Michael-additions and Diels–Alder reactions in water.^176^

**Fig. 19:**
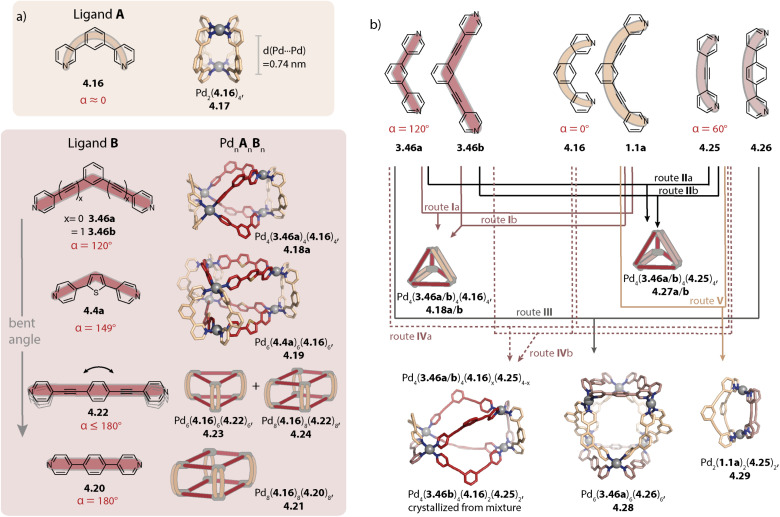
(a) Integrative self-sorting to heteroleptic Pd_*n*_A_*n*_B_*n*_-type assemblies (*n* = 4, 6, 8) through the combination of small ligand 4.16 with collinear binding vectors with ligands 3.46, 4.4a, 4.22, and 4.20 possessing divergent vectors. The bent angle and the flexibility of the latter control the nuclearity. (b) Overview of heteroleptic assembly formation with binary or ternary mixtures of ligands with collinear vectors (1.1a and 4.16), 60° binding angles (4.26 and 4.25), or 120° angles (3.46). Tetrahedral Pd_4_A_4_B_4_ can be obtained from 0°-bent angle ligand 4.16 with 120°-bent angle ligands of different lengths (route I) or upon combination of one of the latter two with 60°-bent angle ligand 4.25 (route II). Tetrahedral Pd_4_A_4_B_*x*_C_4−*x*_ can be obtained when ligands 3.46a or 3.46b are combined with ligands 4.16 and 4.25. Trigonal antiprismatic assembly 4.28 is obtained with ligands 4.16 and 4.25. Ligands 1.1a and 4.25 self-assemble to form dinuclear cage 4.29.

In 2021, Severin and coworkers achieved selective assembly of Pd_4_A_4_B_4_-type tetrahedral, Pd_6_A_6_B_6_-type trigonal prismatic, and Pd_8_A_8_B_8_-type tetragonal prismatic architectures by variation of the binding angle of one of the employed ligands. Heteroleptic assembly formation was investigated with ligand 4.16, possessing collinear binding vectors. The ligand is predisposed for the formation of heteroleptic assemblies as its homoleptic cage Pd_2_(4.16)_4_, 4.17 is strained due its small Pd⋯Pd distance, amongst other factors (Fig. 19a). The combination of 4.16 with ligand 3.46a, possessing a bent angle of 120° led to selective formation of pseudo-tetrahedral Pd_4_(4.16)_4_(3.46a)_4_, 4.18a. In accordance with the binding modes observed in homoleptic tetrahedra, more open ligand 3.46a occupies the singly bridged edges while two ligands 4.16 form macrocyclic motif Pd_2_(4.16)_2_. Interestingly, a similar outcome was achieved when alkyne-spaced ligand 3.46b was employed; however, ligand 4.16 could not be replaced by its alkyne spaced analogue 1.1a. This apparent disparity can be explained with the higher strain of homoleptic 4.17 in comparison to the longer analogue 1.2a. The combination of wider ligand 4.4a, based on thiophene, with ligand 4.16 resulted in the formation of a trigonal prismatic cage Pd_6_(4.4a)_6_(4.16)_6_, 4.19. Similar to the assembly 4.15 reported by Mukherjee and coworkers, two counts of ligand 4.16 form macrocyclic moieties Pd_2_(4.16)_2_ that are bridged by ligands 4.4a. Next, the authors further increased the binding angle of the second ligand by applying linear ligands in which the *para*-pyridines were either bridged by a phenyl (4.20) or by a *para*-diethynylbenzene linker (4.22). From a purely geometrical point of view, the combination of a linear ligand with ligand 4.16 would lead to the formation of a tetragonal prismatic (cuboid) assembly. Indeed, the exclusive formation of Pd_8_(4.16)_8_(4.20)_8_, 4.21 was observed for short, and thus rigid, ligand 4.20. Conversely, the alkyne linkers endow ligand 4.22 with enough flexibility to allow for the formation of entropically favoured trigonal prism Pd_6_(4.16)_6_(4.22)_6_, 4.23 along with tetragonal prism Pd_8_(4.16)_8_(4.22)_8_, 4.24.^156^

Tetrahedral assemblies can be obtained upon the combination of 120° bent angle ligands 3.46 with ligand 4.16 (as just discussed, Fig. 19b, route I). Ligand 4.16 occupying the doubly-bridged edges can be replaced by ligand 4.25, possessing an angle of 60° (route II). Excitingly, the combination of the two phenyl-spaced ligands 3.46a and 4.26 led to novel species Pd_6_(3.46a)_6_(4.26)_6_, 4.28, a trigonal antiprismatic assembly (route III). Severin and coworkers accomplished this discovery by screening of a virtual combinatorial library. More precisely, the authors combined a variety of well-known ligands (1.1a, 1.8, 2.24, 2.55, bis-pyridyl analogue of 3.1, 3.46a) with substoichiometric amounts of a Pd(ii) salt in order to favour formation of particularly stable assemblies. Examination of the X-ray crystal structure reveals that the peculiar ligand arrangement in 4.28 allows for a nearly ideal square planar coordination geometry of the Pd(ii) nodes. For examining the generality of the assembly of hexanuclear heteroleptic cages, the authors employed ligands with similar angles albeit with different lengths and bulk. Hence, instead of ligand 4.26, clathrochelate ligand 2.46d was employed and ligand 3.46a was replaced by an analogue with similar clathrochelate moieties between the 1,3-benzene-core and the *para*-pyridines. Interestingly, this led to the formation of a hexanuclear prismatic architecture instead of an antiprismatic one as observed in 4.28.^177^ In another vein, the authors showed that ligand 1.1a assembles with ligand 4.25 to form dinuclear cage *cis*-Pd_2_(1.1a)_2_(4.25)_2_, 4.29. The tilted PdN_4_ planes point towards shape complementarity between the two ligands. This is achieved by the bending of the ligands owing to their flexible alkyne spacers. In order to further increase the complexity of the system, the authors aimed at incorporating all three kinds of ligands (*i.e.* with 0°, 60°, and 120° bent angles) within one assembly. For this, the self-assembly was examined with the various ligand combinations (eight combinations and different ratios). Solely the combination of ligands 4.16 and 4.25 with either 3.46a or 3.46b afforded assemblies comprising all three ligands (routes IVa and IVb). More specifically, tetrahedra Pd_4_(3.46a/b)_4_(4.16)_*x*_(4.25)_4−*x*_ were obtained in which 120° ligand 3.46 occupies the singly bridged edges while ligands 4.16 and 4.25 are statistically distributed over the doubly bridged edges.^208^

Driven by the aim of preparing cages that carry multiple functionalities, vast progress has been achieved in the development of strategies for designing Pd(ii)-based heteroleptic cages in the last decade. For favouring a heteroleptic coordination environment in dinuclear cages, the donor sites can be equipped with steric bulk or functionalities that allow for attractive secondary interactions (coordination sphere engineering, CSE). Alternatively, repulsive or attractive interactions are installed in more central ligand positions, as in the adjacent backbone interaction (ABI) approach. In other examples, cavity-filling steric bulk is employed to steer the self-sorting outcome. The necessity for such ancillary groups can be omitted when suitably shaped ligands are used in the shape complementary assembly (SCA) strategy. The combination of different strategies was shown to allow for accessing dinuclear cages with up to four differentiable ligands. Similar strategies have been employed for designing heteroleptic cages of higher nuclearity. In this case, exohedral steric bulk was also added to control selective self-sorting. For *n* = 6 and *n* = 8, some topologies were accessed that could never be observed when only one kind of ligand was employed, namely trigonal prismatic and antiprismatic as well as tetragonal prismatic assemblies. In contrast to dinuclear cages, defined heteroleptic assemblies of higher nuclearity which carry more than two kinds of ligands have so far rarely been reported and thus pose a challenge for future investigations.

## 5. Orientational self-sorting

**Fig. 20:**
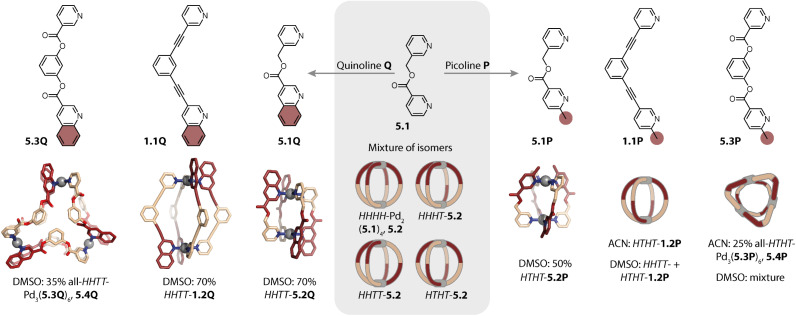
Biasing the self-assembly of asymmetric ligands by introducing steric constraints on the donor site. (Left) For quinoline-equipped ligands the *HHTT*-isomer is favoured in DMSO. (Middle) Bis-pyridyl ligand 5.1 assembles to form a mixture of different isomers. (Right) For picolyl-equipped ligands, there is a bias towards *HTHT*-isomers.

An alternative approach for accessing Pd_*n*_L_2*n*_ assemblies of higher structural complexity is the use of ligands lacking the typical two-fold symmetry seen in all ligands discussed so far. On the one hand, this method can give rise to assemblies with Pd(ii) ions sitting in differentiable coordination environments. On the other hand, it permits for the partitioning of functional groups within the cavity, holding promise for cages capable of binding low-symmetry guests. Most of the work reported so far focuses on the smallest family member, the Pd_2_L_4_ cage, where four isomers can be obtained depending on the relative arrangement (*H*: head and *T*: tail) amongst the ligands (Fig. 20, middle). Significant progress regarding the orientational self-sorting of asymmetric ligands was achieved in recent years by the groups of Lewis,^179,187,189,193,197^ Chand,^191,192,194^ and others^180,181,186,188,196^ from the experimental side and the group of Jelfs^179,187,189^ from the theoretical side.

In 2020, Chand and coworkers showed that ligand 5.1, possessing an asymmetrical linker between the two pyridines, assembles to form a mixture of different isomers Pd_2_(5.1)_4_, 5.2 (Fig. 20, middle).^178^ Later, the ligand design was picked up by Lewis, Jelfs, and coworkers who substituted one of the donor moieties with sterically more demanding picolyl (5.1P) or quinolinyl (5.1Q) moieties. Interestingly, the picolyl-based ligand 5.1P shows a preference for the *HTHT*-5.2P isomer, while self-assembly of quinoline-based ligand 5.1Q leads to a higher fraction of *HHTT*-5.2Q isomer when DMSO is used as the solvent. Addition of acetonitrile led to an increase in the fraction of *HTHT*-5.2 for both ligands. Supported by DFT-computations, the authors suggested that the *HTHT*-5.2 isomer is favoured from a purely structural point of view while the *HHTT*-isomer offers a suitable pocket for binding of H-bond acceptor solvents such as DMSO. The latter effect is of particular importance for the ligand with quinoline donors due to the polarized quinoline proton. Pleasingly, this behaviour was also observed for the self-sorting of ligands 1.1Q and 1.1P: the former shows a preference for the *HHTT*-1.2Q isomer while the latter assembles to form *HTHT*-1.2P in acetonitrile and to form a mixture of the two isomers in DMSO. Ligands 5.3Q and 5.3P assemble to form three-membered rings Pd_3_(5.3)_6_, 5.4. Similar effects lead to a preference for all-*HHTT*-Pd_3_(5.3Q)_6_, 5.4Q in the case of 5.3Q in DMSO and for all-*HTHT*-Pd_3_(5.3P)_6_, 5.4P in the case of 5.3P in ACN.^179^

**Fig. 21:**
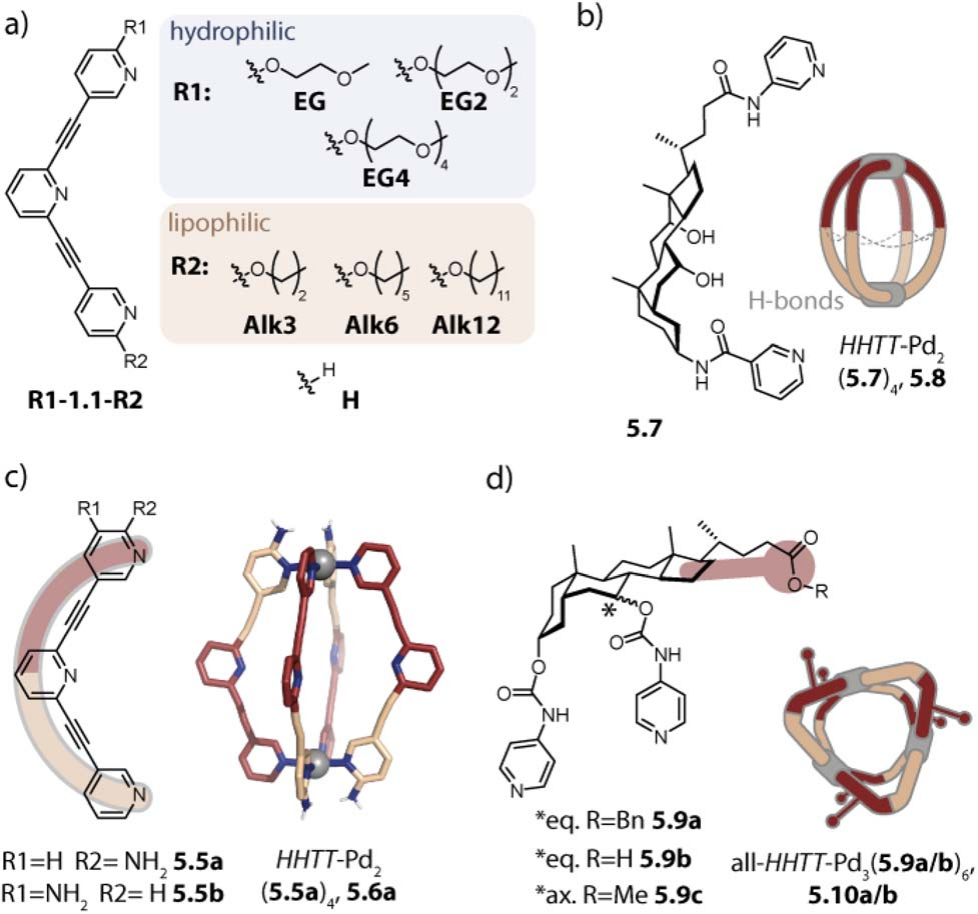
(a) Design of ligands for an attempt to steer orientational self-sorting through dispersive interactions. (b) Self-sorting of ligand 5.7 to *HHTT*-5.8 through backbone-centred H-bonds. (c) Steering self-sorting through H-bonds between amino substituents on the donor sites. (d) Ligands 5.9 and selectively formed all-*HHTT* isomer 5.10.

In 2023, Lusby, Crowley, and coworkers investigated the effect of attractive interactions within the coordination sphere on the orientational self-sorting. A selection of ligands, termed here R1-1.1-R2 (Fig. 21a), with variable hydrophilicity has been synthesized: ligands H-1.1-Alk3, H-1.1-Alk6, and H-1.1-Alk12 are equipped with a hydrophobic alkoxy chain on one of the pyridines and ligands EG-1.1-H, EG2-1.1-H, and EG4-1.1-H possess hydrophilic ethylene glycol chains of increasing length. Furthermore, ligands EG-1.1-Alk3, EG2-1.1-Alk6, and EG4-1.1-Alk12 have been synthesized which are functionalized with alkoxy chains on one ligand end and ethylene glycol chains on the other end that are of varying length. Intuitively, DFT computations supported that for the EG-1.1-Alk ligands, the *HHHH*-Pd_2_(1.1)_4_ isomer, in which hydrophobic and hydrophilic chains are segregated, should be favoured. A similar isomer was favoured for H-1.1-Alk ligands, while the *HTHT*-isomer was predicted to be the most stable for the EG-1.1-H-type ligands. The predicted energy differences between the isomers were, however, small. In accordance with this, the authors observed the formation of mixtures of isomers of Pd_2_(1.1)_4_ when the ligands were combined with Pd(ii). Hence, dispersion interactions were not sufficient here for steering the self-sorting towards a single isomer. The authors also equipped one of the ligand ends with 2-amino pyridine. As discussed in Section 4.1, this strategy allowed for the formation of (kinetically stable) *cis*-Pd_2_A_2_B_2_ with the corresponding symmetrical ligands A and B. In a similar fashion, ligand 5.5a assembled to form *HHTT*-Pd_2_(5.5a)_4_, 5.6a (Fig. 21c). DFT computations supported that the orientational self-sorting was steered by H-bonding interactions between neighbouring amino groups and that the isomer was, similar to heteroleptic 3.6, a kinetic product. When the amino groups were positioned farther away from the nitrogen donor, more precisely in the *meta*-position for ligand 5.5b, a mixture of isomers was obtained, highlighting the importance of the intramolecular H-bonds for the selectivity.^180^

H-bonding interactions for driving orientational self-sorting were also exploited by Natarajan and coworkers. In 2019, the authors reported dinuclear cages Pd_2_(5.7)_4_, 5.8 formed by ligand 5.7 which is based on cholic acid and possesses two hydroxyl groups on its flanks (Fig. 21b). The latter allows for inter-ligand hydrogen bonds in the helically twisted *HHHH*-5.8 isomer. These interactions were vital for selectivity since removal of one or both hydroxy groups resulted in the formation of a mixture of isomers.^181^ Further recent reports by McTernan as well as Palma and coworkers on the stereoselective cage assembly from peptide-based bis-pyridyl ligands are noteworthy.^182,183^

Rissanen and coworkers reported in 2015 on the self-assembly of bile acid–based ligand 5.9a, having a multitude of chiral centres, to a single isomer of three-membered ring Pd_3_(5.9a)_6_, 5.10a (Fig. 21d). NMR experiments in combination with DFT computations and chloride-induced disassembly studies point towards an all-*HHTT* arrangement of the ligands in 5.10a.^184^

In a later study, the authors employed ligand 5.9b, which possesses, in contrast to ligand 5.9a a free carboxyl group. Assembly Pd_3_(5.9b)_6_, 5.10b could only be obtained through stepwise synthesis by first adding a Fe(III) or Cu(ii) salt, followed by a transmetalation with Pd(ii). Supported by ion mobility mass spectrometric experiments, the authors suppose that the ligands are arranged in an all-*HHTT* fashion, similarly to 5.10a. The unprotected carboxyl group renders 5.10b water soluble and amphiphilic. The authors also observed higher order self-assembly: addition of water to the cage (synthesized by transmetalation of the Cu(ii) assembly) in DMSO led to aggregation and gel formation. Furthermore, investigation with transmission electron microscopy revealed evaporation-induced formation of hexagonal particles.^185^

Very recently, some of the authors incorporated a bile acid which is an epimer to 5.9a/b, into bis-pyridyl ligand 5.9c. Importantly, since the pyridine moiety is attached *via* the epimeric hydroxy group, ligand 5.9c has an altered binding angle. Assembly of 5.9c with Pd(ii) led to a mixture of Pd_3_(5.9c)_6_, Pd_4_(5.9c)_8_, Pd_5_(5.9c)_10_, and Pd_6_(5.9c)_12_ species.^186^

**Fig. 22:**
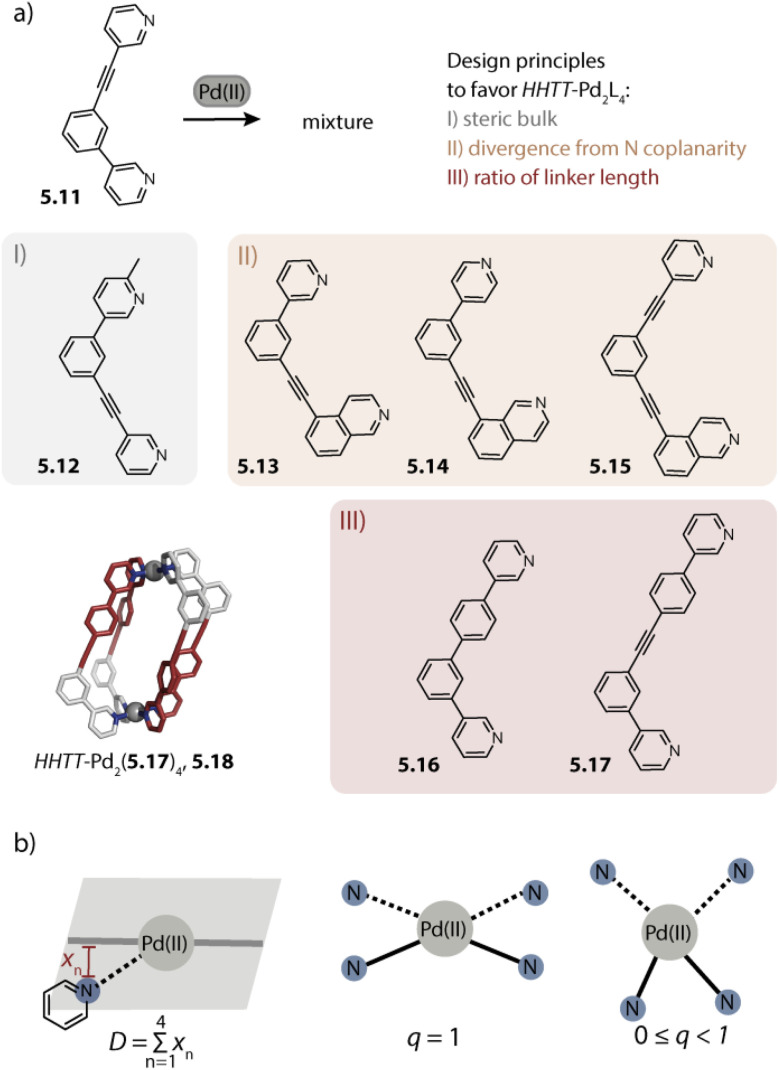
(a) Modification of ligand 5.11 by using (I) steric bulk on the donor site, (II) alteration of the offset between the nitrogen donor groups, and (III) increase in the difference in the linker lengths for steering orientational self-sorting to the *HHTT*-Pd_2_L_4_ isomer and crystal structure of *HHTT*-5.18. (b) Illustration of heuristics *D*, the maximal sum of the deviations of the Pd(ii)–nitrogen bonds from the average PdN_4_ plane and *q*, the minimum square planar order parameter for estimating the stability of cage isomers.

Lewis and coworkers showed in 2020 that the asymmetry of ligand 5.11, in which the two ligand arms differ only in one alkyne spacer, is not sufficient for steering orientational self-sorting to one defined species (Fig. 22a). More precisely, the *HHTT*- and the *HHHT*-isomer were predicted to be relatively similar in energy. The bias for the *HHTT*-isomer could be sufficiently increased by endowing one end of the ligand with a picolyl donor moiety (5.12). Hence, orientational self-sorting was achieved here through a combination of steric and geometrical constraints (principle I). In another fashion, selectivity for the *HHTT*-isomer was achieved by increasing the geometrical constraints: in 5.13 and 5.14, the planes orthogonal to the donor vectors are parallel but have, however, a larger offset as compared to ligand 5.11 (principle II). In both cases, the *HHTT*-isomer was obtained within two hours at room temperature. Ligand 5.15 differs from ligand 5.13 in the alkyne spacer only, which increases the similarity between the two arms. Selective self-assembly to the *HHTT*-isomer was observed; however, the equilibration time was significantly longer as compared to the one for the self-assembly of ligands 5.13 and 5.14.^187^

Zhang, Wang and coworkers increased the asymmetry of ligand 5.11 by increasing the length of one linker (principle III). Self-assembly with phenyl spaced ligand 5.16 afforded *HHTT*-Pd_2_(5.16)_4_ as the main species along with traces of other species. A further increase in ligand dissymmetry by incorporating a phenyl–alkynyl-spaced linker in 5.17 afforded the same isomer *HHTT*-Pd_2_(5.17)_4_, 5.18 as the sole species. DFT computations support that by increasing the linker length, the energy difference between the *HHTT*- and the *HHHT*-isomer increases (5.11 < 5.16 < 5.17).^188^

In a combined theoretical and experimental study, Jelfs, Lewis, and Tarzia developed a workflow that aims at predicting the selective orientational self-sorting to *HHTT*-Pd_2_L_4_ cages with high throughput. For ranking the stability of the isomers of a virtual library of asymmetric ligands, three heuristics were considered: the energy difference Δ*E* between the two most stable isomers, as well as the sum of the deviations of the Pd(ii)–nitrogen bonds from the average PdN_4_ plane *D* and the minimum square planar order parameter *q* (in an ideal square planar environment *D* = 0 and *q* = 1, Fig. 22b). From the isomers that were predicted to have parameters favouring the formation of a single *HHTT*-isomer (Δ*E* ≥ 6 kJ mol^−1^, *D* < 0.1 Å, *q* > 0.95), three were already shown to selectively form the *HHTT*-isomer (ligands 5.13, 5.14, and 5.15). For testing the fidelity of the approach, the authors experimentally investigated the self-assembly of five new ligands. While single isomers were obtained for ligands strictly obeying the set structural parameters, the approach was less reliable for small Δ*E* values. Hence, the parameters considered do not seem to describe all factors influencing the orientational self-sorting. Omitted effects include the templation by anions or solvents as well as ligand flexibility.^189^

**Fig. 23:**
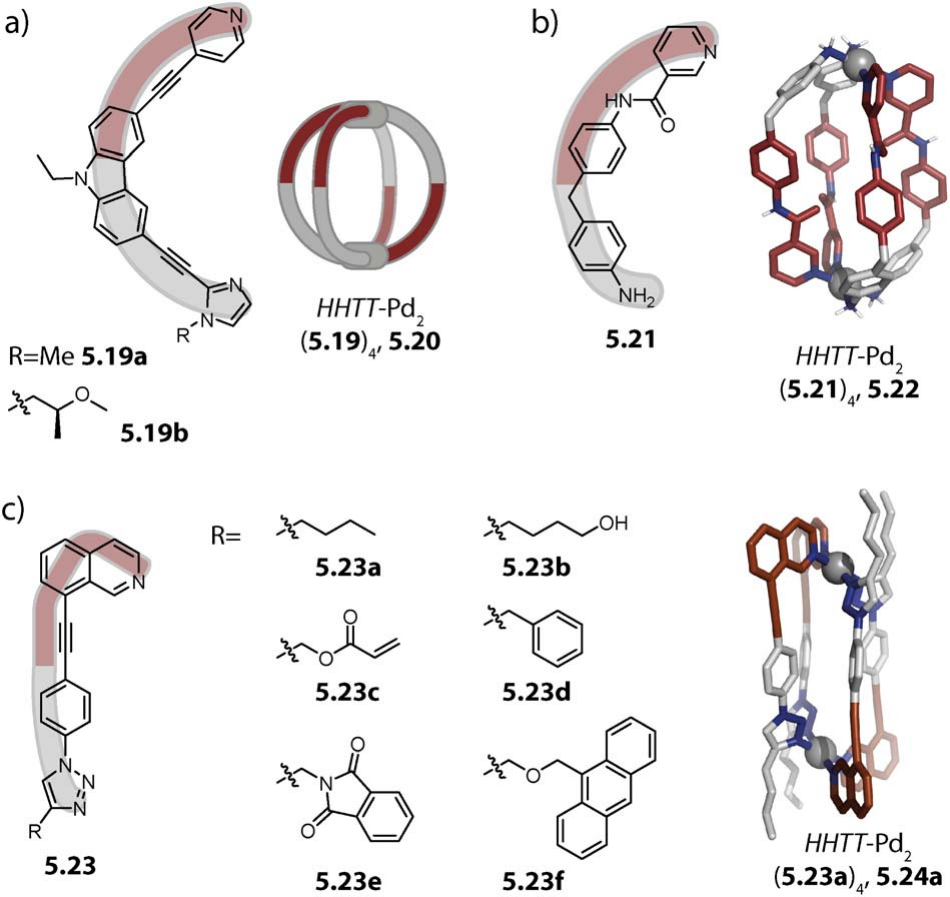
Orientational self-sorting with ligands bearing differing donor moieties. (a) Ligand 5.19 combines pyridine and imidazole donors. (b) Ligand 5.21 comprises pyridine and aniline donors. (c) Ligand 5.23 has isoquinoline and triazole donors.

In 2019, Ogata and Yuasa studied the self-assembly of ligand 5.19 which is equipped with a *para*-pyridine as well as an imidazole donor moiety. When 5.19 and the Pd(ii) source are combined in a 2 : 1 ratio, a single isomer Pd_2_(5.19)_4_, 5.20 was obtained (Fig. 23a). NMR spectroscopic investigations in combination with DFT computations support that the *HHTT*-isomer is favoured. Steric repulsion between pyridine and imidazole protons caused a twisting of the assembly which led to the existence of two diastereomers if chiral ligand 5.19b was employed. The authors furthermore exploited the superior donor strength of imidazole: upon decreasing the 5.19: Pd(ii) stoichiometry, open structure Pd_1_(5.19)_4_ was obtained, where the ligand only coordinates *via* the imidazole end. The structural conversion was accompanied by the release of an encapsulated anion.^190^

The group of Chand employed ligand 5.21 with 4-aniline and *meta*-pyridine ends. The self-assembly was studied with various Pd(ii) precursors using DMSO as the solvent. In each case, formation of a single isomer assigned to *HHTT*-Pd_2_(5.21)_4_, 5.22 was achieved, noteworthily with the aniline amino substituents acting as donors besides the pyridines (Fig. 23b). Furthermore, robust selectivity for this isomer was observed at different temperatures as well as at varying concentrations. The authors performed DFT computations and molecular dynamics (MD) studies which supported that the superior stability of the *HHTT*-isomer is driven on one hand by its least conformational strain and on the other hand by stabilization through electrostatic and van-der-Waals interactions.^191^

Additionally, the same group reported an asymmetric ligand with *meta*-pyridine and 3-aniline donor groups separated by an amide bond. This short ligand was shown to assemble to form a single isomer Pd_3_L_6_ which was, based on spectroscopic and computational investigations, assigned to the all-*HHTT* isomer.^192^

Lewis presented in 2021 the selective formation of *HHTT*-Pd_2_(5.23)_4_, 5.24 isomers with ligands 5.23, terminated with an isoquinoline and a triazole donor (Fig. 23c). The latter was introduced in the last step *via* a CuAAC click reaction, allowing for easy functionalization of the ligand. While the selectivity for the *HHTT*-isomer proved robust across a variety of different electronic situations for the triazole donor (ligands 5.23a–f), variation of the donor strength affected the self-assembly when ligand and Pd(ii) were combined in a 4 : 1 ratio. For phthalimide-appended ligand 5.23e, a mononuclear Pd_1_(5.23e)_4_ complex was observed, similar to the work of Ogata and Yuasa.^190^ The ligand coordinates here solely *via* the isoquinoline donor moieties as the substituent in 5.23e (further) reduces the triazole donor strength. In contrast, when ligand 5.23a was employed, a mixture of free ligand, mono-, and dinuclear species was observed. Exploiting the isostructural nature of ligands 5.23, the author showed that statistical mixtures of homo- and heteroleptic cages were obtained when multiple ligands were combined.^193^

**Fig. 24:**
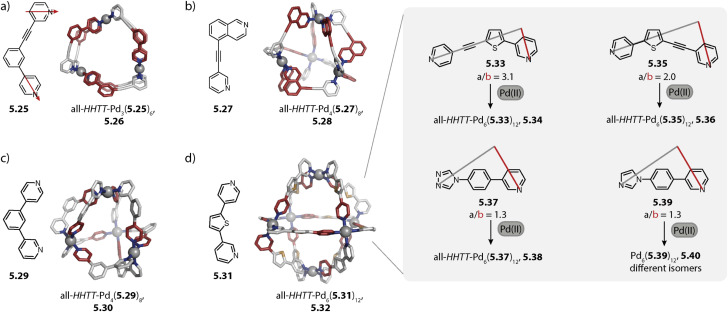
Orientational self-sorting to the all-*HHTT* isomer in tri- (a), tetra- (b and c), and hexanuclear species (d). The inset shows the selectivity of the orientational self-sorting for ligands with differing ratios *a*/*b*.

Zhang and coworkers substituted the *meta*-pyridine in ligand 5.11 with a *para*-pyridine resulting in an increased binding angle of ligand 5.25 (Fig. 24a). In accordance with the trends observed for homoleptic cages (Chapter 2), this resulted in the formation of a species of higher nuclearity, here a three-membered ring. Noteworthily, geometric constraints prompted selective formation of all-*HHTT*-Pd_3_(5.25)_6_, isomer 5.26.^188^

Recently, Chand and coworkers reported the formation of a tetrahedral all-*HHTT*-Pd_4_(5.27)_8_, 5.28 by applying geometrically constrained ligand 5.27 with a bent angle of approximately 120° (Fig. 24b).^194^

In 2021, Severin and coworkers reported tetrahedral and octahedral assemblies with asymmetric ligands 5.29 and 5.31 that possess wide bent angles (Fig. 24c and d). The number of possible isomers reaches 35 in the case of the tetrahedral assembly and to 112 in the case of the octahedral one. Astonishingly, the authors observed the formation of single isomers all-*HHTT*-Pd_4_(5.29)_8_, 5.30 and all-*HHTT*-Pd_6_(5.31)_12_, 5.32 in which two different ligand ends are arranged in a *cis*-configuration on each Pd(ii) centre.^195^ The preferred orientation of the ligands can be traced back to geometrical constraints, as was explored in a later study by the same authors on the basis of octahedral assemblies: ligand 5.31 can be characterized by the ratio of the two donor vectors *a*/*b*. Two ligands opposing each other in the assembly can either be oriented *HH*, *TT*, or *HT*. Only the latter case allows for a Pd–Pd–Pd angle of 90° required for an octahedron. Conceivable limitations of the so-called *cis*-rule are a small *a*/*b* ratio as well as ligand flexibility. Both aspects were altered in alkyne-spaced ligands 5.33 and 5.35. Ligand 5.33, possessing a large *a*/*b* ratio cleanly assembles, similarly to ligand 5.31, to form octahedron all-*HHTT*-Pd_6_(5.33)_12_, 5.34. When ligand 5.35 with a decreased asymmetry was employed, prolonged heating was required for the selective formation of all-*HHTT*-Pd_6_(5.35)_12_, 5.36. The approximate rectangular bent angle that allows for the formation of an octahedron is maintained in ligands 5.37 and 5.39, possessing triazole or imidazole donors, respectively. The former strictly follows the *cis*-rule, yielding all-*HHTT*-Pd_6_(5.37)_12_, 5.38. In contrast, a mixture of isomers Pd_6_(5.39)_12_, 5.40 was obtained with ligand 5.39, that differs merely in its electronic situation. The selectivity might be hampered by the formation of kinetically trapped intermediates due to the higher basicity of the imidazole donor moiety, as hypothesized by the authors.^196^

**Fig. 25:**
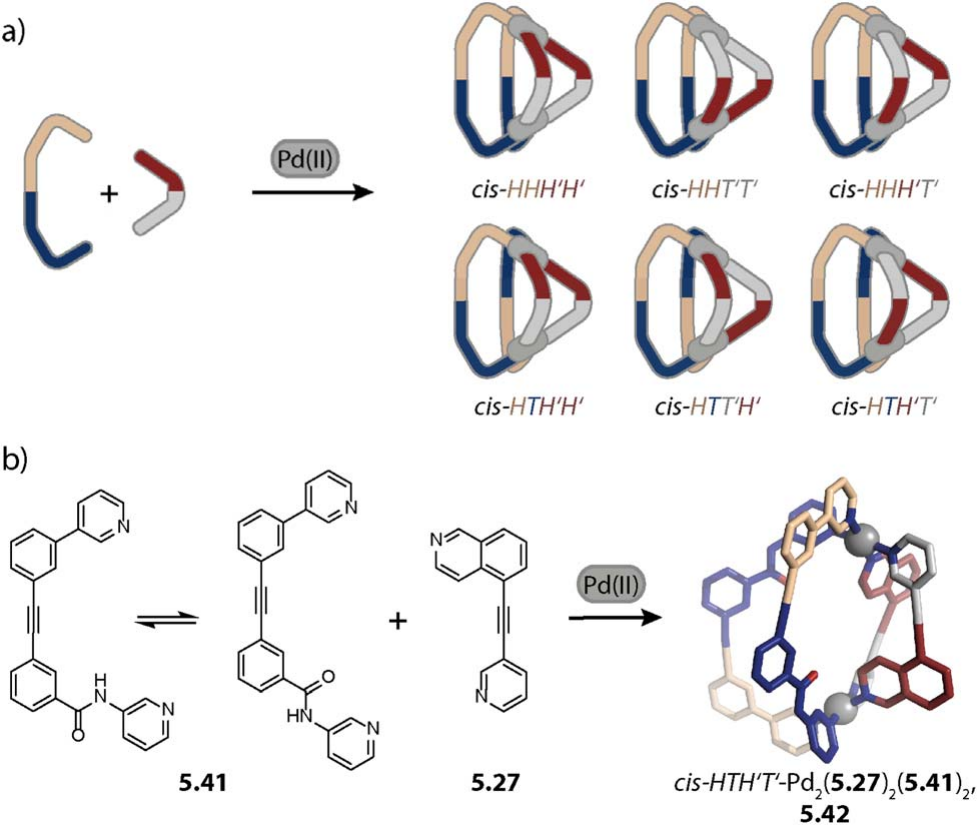
(a) Possible isomers for the self-assembly of two asymmetric, shape-complementary ligands in the absence of factors driving orientational self-sorting (enantiomers are not shown). (b) Selective assembly of *cis-HTH*′*T*′-5.42 through the SCA approach in combination with geometric constraints driving orientational self-sorting.

Only very recently, Chand and coworkers were able to combine the non-statistical self-assembly of heteroleptic cages with orientational self-sorting. For this, they employed two strategies: the SCA approach for forming *cis*-Pd_2_A_2_B_2_ cages as well as geometrical constraints for driving orientational self-sorting. For the former, the two ligands 5.27 and 5.41 were designed in a way that their donor vectors are convergent and divergent, respectively (for ligand 5.41 in both conformations, Fig. 25a). The geometric complementarity of 5.27 and 5.41 reduced the number of possible isomers from 31 to six. Furthermore, the structural constraints were expected to only allow a ligand arrangement in which the amido-pyridine ends of 5.41 oppose the pyridine ends of ligands 5.27 and the pyridine ends of 5.41 oppose the isoquinoline ends of 5.27. This leaves two possible isomers, namely *HHH*′*H*′-Pd_2_(5.27)_2_(5.41)_2_ and *HTH*′*T*′-Pd_2_(5.27)_2_(5.41)_2_. The authors found that *HTH*′*T*′-Pd_2_(5.27)_2_(5.41)_2_, 5.42 was formed selectively (Fig. 25b). The preference for the antiparallel arrangement of the ligands can be explained with increased steric strain when the two isoquinoline donors of 5.27 sit in *cis*-position to each other. Interestingly, substitution of the alkyne spacer in 5.27 by a phenyl spacer hampered self-sorting to a defined species. It was hypothesized that this is due to a length mismatch.^194^ Lewis and coworkers, on the other hand, achieved isomer-selective assembly of low-symmetry heteroleptic Pd_2_LA_2_LB_2_ cages through a combination of shape-complementary assembly and coordination sphere engineering.^197^

The repertoire of self-assemblies with asymmetric ligands has greatly expanded within recent years. In most cases, ligands with roughly parallel binding vectors have been employed for obtaining dinuclear Pd_2_L_4_ cages, which possess the smallest number of possible isomers. Additionally, all-*HHTT* isomers of higher nuclearity species have been reported, that are three-membered rings, tetrahedra, and octahedra. The development of strategies for orientational self-sorting was highly shaped by already existing ones for the non-statistical self-sorting of heteroleptic cages, that are strategies exploiting steric or geometric constraints or a combination thereof. This showcased that the design principles are transferrable; hence, it is expected that the two areas will mutually benefit from each other in the future. Furthermore, orientational self-sorting allows for differentiable coordination environments; however, the number of examples for species dealing with the consequences of this phenomenon remains scarce. Recently, strategies for integrative and orientational self-sorting have been transferred to Pt(ii) cage chemistry by Preston, using a specific Pt(ii) precursor that ensures sufficient kinetic accessibility of the thermodynamic products in the assembly reaction.^198^

## 6. Chiral cages

Chirality in metallo-supramolecular assemblies can be a consequence of the spatial arrangement of the components upon assembly formation or can stem from the inherent chirality of one or more components. The former, commonly referred to as a “soft” approach is promising for stimuli responsive systems due to its dynamicity. Chiral interaction partners, such as guests, can then transfer their chirality on the assembly *via* non-covalent interactions. The latter, “hard” approach can be advantageous for functional materials, *e.g.* for chiral sensing or asymmetric catalysis, due to its robustness.^199^ Employing chiral ligands as a racemic mixture endows the self-assembly process with a further layer of complexity: in the absence of self-sorting, a mixture of different diastereoisomers can be obtained. Narcissistic chiral self-sorting occurs under self-recognition, leading to a mixture of two assemblies bearing only ligands of the same handedness. In contrast, social chiral self-sorting is based on self-discrimination and may lead to overall achiral *meso*-compounds.

The groups of Kubik and Lützen as well as our group used chiral, peptide-based ligands for assembling three-membered rings^200^ and doubly interlocked lemniscates.^98^ Furthermore, Lützen and coworkers reported homochiral tetrahedra, cubes, and dodecanuclear spheres based on BINOL ligands.^124,127^ Liu and coworkers utilized the BINOL-based cube for obtaining CPL-active host–guest complexes with BODIPY guests.^201^ The group of Zysman-Colman formed three- and four-membered rings for photophysical investigations based on paracyclophane^202^ and homochiral Ir(iii)^203^ complexes. Furthermore, Rissanen and coworkers reported a series of assemblies with ligands based on bile acids,^184,185^ as described in Chapter 5. Aside from these architectures that were assembled from enantiomerically pure ligands, the chiral self-sorting of racemic mixtures of ligands gained particular attention and will be the focus of this chapter.

**Fig. 26:**
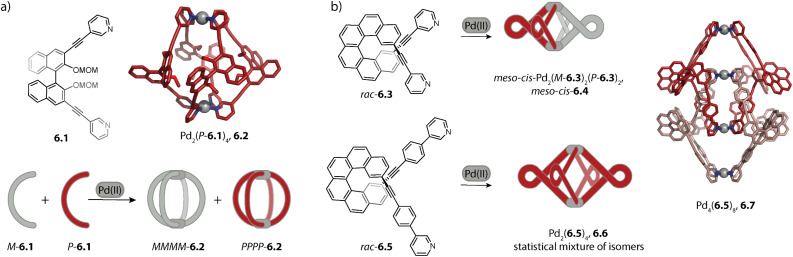
(a) Narcissistic chiral self-sorting to a pair of enantiomeric lantern-shaped cages 6.2. (b) Social chiral self-sorting to *meso-cis*-6.4 that is lost upon prolongation of the linker.

Lützen and coworkers reported in 2013 on the first system showing narcissistic chiral self-sorting in Pd_2_L_4_ cages. The authors studied the self-assembly of BINOL-based ligands *M*- or *P*-6.1 possessing axial chirality. When 6.1 is employed as a racemic mixture, six different isomers can potentially be obtained: two homochiral cages, two achiral, diastereomeric cages (*meso-cis* and *meso-trans*), and two cages carrying both enantiomers in a 3 : 1 ratio. Self-assembly occurred under chiral self-recognition, leading to a narcissistic mixture of the two homochiral cages Pd_2_(*M*-6.1)_4_, *MMMM*-6.2 and Pd_2_(*P*-6.1)_4_, *PPPP*-6.2 (Fig. 26a).^204^

Recently, we reported the narcissistic chiral self-sorting of Tröger-based ligands *M*-L and *P*-L into Pd_2_(*M*-L)_4_ and Pd_2_(*P*-L)_4_ capsules upon encapsulation of fullerene guests or upon crystallization, respectively.^78^

Social chiral self-sorting in Pd_2_L_4_ cages, on the other hand, was reported by us in 2019 for the first time. Ligands *M*- and *P*-6.3, based on a helicene backbone, assemble with Pd(ii) to form dinuclear homochiral cages Pd_2_(*M*/*P*-6.3)_4_, *MMMM*/*PPPP*-6.4. When a racemic mixture of the ligand is employed, self-discrimination to *meso-cis*-Pd_2_(*M*-6.3)_2_(*P*-6.3)_2_ and *meso-cis*-6.4 is observed (Fig. 26b). Interestingly, a breakdown of chiral self-sorting was observed for ligands *M*- and *P*-6.5, differing only in the elongated linker between the helicene core and the pyridine donors. This was thought to originate from the increased distance between the chiral backbones in the assembly. The enantiopure monomeric cages Pd_2_(*M*/*P*-6.5)_4_ and *MMMM*/*PPPP*-6.6 were shown to discriminate between guests of opposite chirality. Furthermore, in line with the criteria for the formation of interlocked double cages (Chapter 2), elongated ligand 6.5 assembled in acetonitrile to form double cage Pd_4_(6.5)_8_, 6.7.^134^

**Fig. 27:**
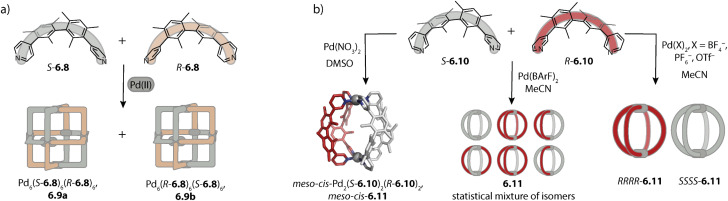
(a) Social chiral self-sorting to a pair of cube enantiomers 6.9. (b) Solvent- and counter anion-dependent chiral self-sorting with a racemic mixture of ligands 6.10.

Recently, the group of Natarajan observed social chiral self-sorting in Pd_6_L_12_ cubes. Ligands 6.8 possess axial chirality and a rectangular bent angle. Due to the increased number of components in hexanuclear in comparison to dinuclear cages, the number of possible isomers reaches 145. Excitingly, the authors observed the selective formation of a racemic pair of heterochiral cages Pd_6_(*S*-6.8)_6_(*R*-6.8)_6_, 6.9 (Fig. 27a).^205^

The same group additionally explored the chiral self-sorting with an isomer of 6.8, ligand 6.10 possessing *meta*-pyridine donors. Intriguingly, when BArF^−^ was employed as the counter anion which, owing to its large size, resides outside of the narrow cavity, no chiral self-sorting was observed (Fig. 27b). However, social chiral self-sorting to *meso-cis*-Pd_2_(*S*-6.10)_2_(*R*-6.10)_2_, *meso-cis*-6.11 was observed in the presence of NO_3_^−^ in DMSO and narcissistic chiral self-sorting to the homochiral pair *SSSS*-6.11 and *RRRR*-6.11 with BF_4_^−^, PF_6_^−^ and OTf^−^. Chiral self-sorting was furthermore shown to be solvent-dependent for NO_3_^−^ or BF_4_^−^ as counter anions: with NO_3_^−^, a solvent switch from DMSO to acetonitrile resulted in a mixture of *meso-cis*-6.11 and a pair of homochiral cages. Additionally, a mixture of different isomers was obtained for the self-assembly with BF_4_^−^ in DMSO.^206^

**Fig. 28:**
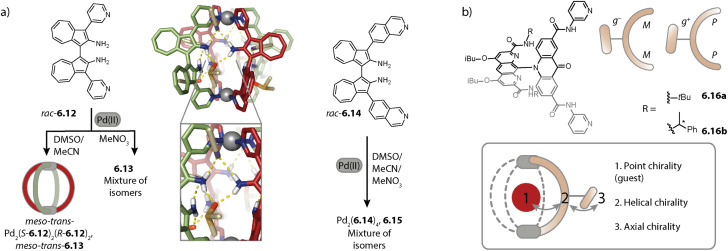
(a) Solvent dependent chiral social self-sorting with biazulene-based ligand 6.12; the expansion of the X-ray structure shows solvent molecules acting as tethers between two adjacent ligands. (b) Bidirectional transmission of chirality between the cavity and the periphery of the cage.

We recently carried out a thorough investigation of the solvent dependency of chiral self-sorting in helical cages with ligands 6.12 and 6.14 that are based on axially chiral 1,1′-biazulene-2,2′-diamine, a structural motif reminiscent of widely used BINOL (with azulene being an isomer of the therein contained naphthalene). Short ligand 6.12 and long ligand 6.14 both assemble to form Pd_2_L_4_ cages; however, a striking difference in chiral self-sorting was observed. In DMSO and acetonitrile, racemic 6.12 selectively forms *meso-trans*-Pd_2_(*S*-6.12)_2_(*R*-6.12)_2_, *meso-trans*-6.13 while a mixture of isomers Pd_2_(6.14)_4_, 6.15 is obtained with racemic 6.14 under the same conditions (Fig. 28a). Close examination of the crystal structure of *meso-trans*-6.13 in combination with DFT computations including explicit solvation reveals that the *meso-trans* isomer is stabilized by individual solvent molecules that connect two adjacent ligands *via* hydrogen bonding. Accordingly, changing the solvent to nitromethane, a significantly weaker H-bond acceptor, resulted in a loss of chiral self-sorting (to be gained back by adding small amounts of DMSO). Such an H-bond tethering is not possible in 6.15 due to the larger distances between the amino groups in the ligand backbones.

Examples for the transfer of chirality from homochiral Pd_*n*_L_2*n*_ cages to encapsulated guests were reported, either giving rise to chiral guest discrimination^134^ or to induced guest chirality.^77,78,201^ Conversely, guest-to-host chirality transfer has been observed in terms of the stabilization of chirality in dynamically chiral hosts through binding of enantiopure guests.^38,46,89^

Very recently, Gan and coworkers reported a bidirectional transmission of chiral information between a guest and the exterior of a helical cage. The authors incorporated in helicates Pd_2_(6.16)_4_ another stereogenic centre: the 1,8-diazaanthracene segments can rotate relative to the ligand backbone, giving rise to different *gauche* conformations (Fig. 28b). Owing to steric constraints, helical and axial chirality are linked; more precisely, a *PP*-conformation of the ligand favours a positive *gauche* conformation (*g*^+^) while a *MM*-conformation favours *g*^−^. Achiral ligand 6.16a assembles to form *meso-cis*-Pd_2_(*MM*-6.16a)_2_(*PP*-6.16a)_2_ incorporating both *gauche* conformations. Encapsulation of l- or d-titrate guests induced a preference for one of the *gauche* conformations, meaning that the chirality is transmitted to the cage periphery. Homo-axial chirality could also be achieved through incorporation of point chirality in the appended segment of ligand 6.16b. Chiral helicates Pd_2_(*MM*-6.16b)_4_ and Pd_2_(*PP*-6.16b)_4_ were shown to discriminate between l- and d-tartrate guests.^207^

Natural or synthetic chiral backbones have been employed for bestowing coordination assemblies of various nuclearities with chirality. We paid particular attention to the chiral self-sorting of racemic mixtures of ligands to either *meso*-cages or a mixture of the two enantiopure cages. Prolongation of the linker can result in a loss of chiral self-sorting due to the increased distance between the ligand backbones. Furthermore, the solvent and the counter anions can have a pivotal effect on the outcome. A possible origin for the former is the specific interactions between solvent molecules and the ligands at the portals of the cage. We also touched upon a recent example in which the soft and hard chirality of helicates was exploited for chirality transmission from or to an encapsulated guest, respectively.

## 7. Conclusion

To conclude, we surveyed the topological diversity of di- to octanuclear assemblies that are accessible upon simple combination of bis-monodentate organic ligands and Pd(ii) cations. The structure of the organic ligand, *i.e.* its geometry and steric demand, plays a key role in the self-assembly outcome. The stiffness of small, aromatic ligands allows them to be simplified as simple geometric units which facilitate structure prediction. In contrast, solvent and counter anions often exert a templation effect on self-assembly with highly flexible ligands, rendering their topologies notoriously more difficult to predict. Long organic ligands allow for mechanical interlocking which can be inhibited by equipping the ligands with endohedral steric bulk. Strategies for increasing structural complexity include the formation of heteroleptic cages with multiple organic building blocks as well as the usage of asymmetric ligands. For steering the self-sorting towards a defined species, the ligands have been endowed with geometric constraints, steric bulk has been installed at the backbone or at the donor moieties, and attractive as well as repulsive inter-ligand interactions have been exploited. Concerning dinuclear heteroleptic cages, the maximum level of self-sorting to provide Pd_2_ABCD cages with four differentiable ligands has been mastered very recently. Several larger heteroleptic architectures have been reported, most of them carrying two different kinds of ligands. Orientational self-sorting with asymmetric ligands flourished in the last couple of years and defined architectures with up to six Pd(ii) nodes have been attained. We have also treated self-sorting effects with chiral ligands; chiral narcissistic and social self-sorting was shown to be highly dependent on solvent and counter anions.

We anticipate that an enhanced understanding of the factors governing the formation of specific topologies as well as the reliability of strategies for selective self-sorting will allow the field to shift progressively towards more and more complex functional nanosystems. On one hand, the assemblies can be harnessed as platforms for combining multiple functional moieties in a combinatorial fashion. The overall assembly properties emerge then from the interplay of the various components, which holds promise for highly selective molecular receptors, catalysts, and drug delivery systems. Another field of application may be energy harvesting materials, *i.e.* in terms of gaining supramolecular control over the morphology of donor/acceptor-based charge separating layers in photovoltaic devices. While examples for functional cages incorporating two kinds of interoperating ligands have been reported (many by our lab), the newly developed strategies for the non-statistical combination of more than two kinds of ligands have not yet been exploited for applicable cages. On the other hand, as an additional layer of complexity, populations of coexisting assemblies have been reported by us very recently. In biological systems, numerous processes occur simultaneously, either in an orthogonal fashion or are closely interconnected. The dynamic nature of the coordination bond as well as the possibility of incorporating responsive moieties can be exploited for creating networks of communicating and coexisting assemblies. This offers great potential for the transmission and transduction of information or energy, which is of interest for the development of smart materials and of the field of systems chemistry.

## Author contributions

L. N. compiled the draft and created the figures. G. H. C. and L. N. both discussed and revised the manuscript.

## Conflicts of interest

There are no conflicts to declare.

## Data Availability

No primary research results, software or code have been included and no new data were generated or analysed as part of this review.
